# Reconstructing the Deep Population History of Central and South America

**DOI:** 10.1016/j.cell.2018.10.027

**Published:** 2018-11-15

**Authors:** Cosimo Posth, Nathan Nakatsuka, Iosif Lazaridis, Pontus Skoglund, Swapan Mallick, Thiseas C. Lamnidis, Nadin Rohland, Kathrin Nägele, Nicole Adamski, Emilie Bertolini, Nasreen Broomandkhoshbacht, Alan Cooper, Brendan J. Culleton, Tiago Ferraz, Matthew Ferry, Anja Furtwängler, Wolfgang Haak, Kelly Harkins, Thomas K. Harper, Tábita Hünemeier, Ann Marie Lawson, Bastien Llamas, Megan Michel, Elizabeth Nelson, Jonas Oppenheimer, Nick Patterson, Stephan Schiffels, Jakob Sedig, Kristin Stewardson, Sahra Talamo, Chuan-Chao Wang, Jean-Jacques Hublin, Mark Hubbe, Katerina Harvati, Amalia Nuevo Delaunay, Judith Beier, Michael Francken, Peter Kaulicke, Hugo Reyes-Centeno, Kurt Rademaker, Willa R. Trask, Mark Robinson, Said M. Gutierrez, Keith M. Prufer, Domingo C. Salazar-García, Eliane N. Chim, Lisiane Müller Plumm Gomes, Marcony L. Alves, Andersen Liryo, Mariana Inglez, Rodrigo E. Oliveira, Danilo V. Bernardo, Alberto Barioni, Veronica Wesolowski, Nahuel A. Scheifler, Mario A. Rivera, Claudia R. Plens, Pablo G. Messineo, Levy Figuti, Daniel Corach, Clara Scabuzzo, Sabine Eggers, Paulo DeBlasis, Markus Reindel, César Méndez, Gustavo Politis, Elsa Tomasto-Cagigao, Douglas J. Kennett, André Strauss, Lars Fehren-Schmitz, Johannes Krause, David Reich

**Affiliations:** 1 Department of Archaeogenetics, Max Planck Institute for the Science of Human History, Jena 07745, Germany; 2 Institute for Archaeological Sciences, Archaeo- and Palaeogenetics, University of Tübingen, Tübingen 72070, Germany; 3 Department of Genetics, Harvard Medical School, Boston, MA 02115, USA; 4 Harvard-MIT Division of Health Sciences and Technology, Boston, MA 02115, USA; 5 Francis Crick Institute, London NW1 1AT, UK; 6 Broad Institute of Harvard and MIT, Cambridge, MA 02142, USA; 7 Howard Hughes Medical Institute, Harvard Medical School, Boston, MA 02115, USA; 8 Dipartimento di Biologia e Biotecnologie, Università di Pavia, Pavia 27100, Italy; 9 Australian Centre for Ancient DNA, School of Biological Sciences and The Environment Institute, Adelaide University, Adelaide, SA 5005, Australia; 10 Department of Anthropology, The Pennsylvania State University, University Park, PA 16802, USA; 11 Institutes of Energy and the Environment, The Pennsylvania State University, University Park, PA 16802, USA; 12 Departamento de Genética e Biologia Evolutiva, Universidade de São Paulo, São Paulo 05508-090, Brazil; 13 UCSC Paleogenomics, University of California, Santa Cruz, Santa Cruz, CA 95064, USA; 14 Department of Human Evolution, Max Planck Institute for Evolutionary Anthropology, Leipzig 04103, Germany; 15 Department of Anthropology and Ethnology, Xiamen University, Xiamen 361005, China; 16 Department of Anthropology, The Ohio State University, Columbus, OH 43210, USA; 17 Instituto de Arqueología y Antropología, Universidad Católica del Norte, San Pedro de Atacama, Región de Antofagasta, Antofagasta CP 1410000, Chile; 18 Institute for Archaeological Sciences, Palaeoanthropology and Senckenberg Centre for Human Evolution and Palaeoenvironment, University of Tuebingen, Tübingen 72070, Germany; 19 DFG Center for Advanced Studies, “Words, Bones, Genes, Tools,” University of Tübingen, Tübingen 72070, Germany; 20 Centro de Investigación en Ecosistemas de la Patagonia, Coyhaique 5951601, Chile; 21 Pontifical Catholic University of Peru, San Miguel, Lima 32, Peru; 22 Department of Anthropology, Michigan State University, East Lansing, MI 48824, USA; 23 Central Identification Laboratory, Defense POW/MIA Accounting Agency, Department of Defense, Joint Base Pearl Harbor-Hickam, HI 96853, USA; 24 Department of Archaeology, Exeter University, Exeter EX4 4QJ, UK; 25 Ya’axché Conservation Trust, Punta Gorda Town, Belize; 26 Department of Anthropology, University of New Mexico, Albuquerque, NM 87131, USA; 27 Center for Stable Isotopes, University of New Mexico, Albuquerque, NM 87131, USA; 28 Grupo de Investigación en Prehistoria IT-622-13 (UPV-EHU), IKERBASQUE-Basque Foundation for Science, Vitoria, Spain; 29 Museu de Arqueologia e Etnologia, Universidade de São Paulo, São Paulo 05508-070, Brazil; 30 Museu Nacional da Universidade Federal do Rio de Janeiro, Rio de Janeiro 20940-040, Brazil; 31 Departamento de Estomatologia, Faculdade de Odontologia, Universidade de São Paulo, São Paulo 05508-000, Brazil; 32 Laboratório de Estudos em Antropologia Biológica, Bioarqueologia e Evolução Humana, Instituto de Ciências Humanas e da Informação, Universidade Federal do Rio Grande, Rio Grande do Sul 96203-900, Brazil; 33 Faculdade de Filosofia Ciencias e Letras, Universidade de São Paulo, São Paulo 05508-080, Brazil; 34 INCUAPA-CONICET, Facultad de Ciencias Sociales, Universidad Nacional del Centro de la Provincia de Buenos Aires, Olavarría 7400, Argentina; 35 Comité Chileno del Consejo Internacional de Monumentos y Sitios, Santiago 8320000, Chile; 36 Field Museum of Natural History, Chicago, IL 60605, USA; 37 Universidad de Magallanes, Punta Arenas 6200000, Chile; 38 Escola De Filosofia, Letras E Ciências Humanas, Universidade Federal de São Paulo, São Paulo 07252-312, Brazil; 39 Servicio de Huellas Digitales Genéticas, School of Pharmacy and Biochemistry, Universidad de Buenos Aires y CONICET, Ciudad Autonoma de Buenos Aires, Junin 954, Argentina; 40 CONICET-División Arqueología, Facultad de Ciencias Naturales y Museo, La Plata 1900, Argentina; 41 Naturhistorisches Museum Wien, Vienna 1010, Austria; 42 German Archaeological Institute, Commission for Archaeology of Non-European Cultures, Bonn 53173, Germany; 43 Centro de Arqueologia Annette Laming Emperaire, Miguel A Salomão, Lagoa Santa, MG 33400-000, Brazil; 44 UCSC Genomics Institute, University of California, Santa Cruz, Santa Cruz, CA 95064, USA

**Keywords:** South America, Central America, population genetics, archaeology, anthropology

## Abstract

We report genome-wide ancient DNA from 49 individuals forming four parallel time transects in Belize, Brazil, the Central Andes, and the Southern Cone, each dating to at least ∼9,000 years ago. The common ancestral population radiated rapidly from just one of the two early branches that contributed to Native Americans today. We document two previously unappreciated streams of gene flow between North and South America. One affected the Central Andes by ∼4,200 years ago, while the other explains an affinity between the oldest North American genome associated with the Clovis culture and the oldest Central and South Americans from Chile, Brazil, and Belize. However, this was not the primary source for later South Americans, as the other ancient individuals derive from lineages without specific affinity to the Clovis-associated genome, suggesting a population replacement that began at least 9,000 years ago and was followed by substantial population continuity in multiple regions.

## Highlights

• Genome-wide analysis of 49 Central and South Americans up to ∼11,000 years old
• Two previously unknown genetic exchanges between North and South America
• Distinct link between a Clovis culture-associated genome and the oldest South Americans
• Continent-wide replacement of Clovis-associated ancestry beginning at least 9,000 years ago

## Graphical Abstract

**Figure:**
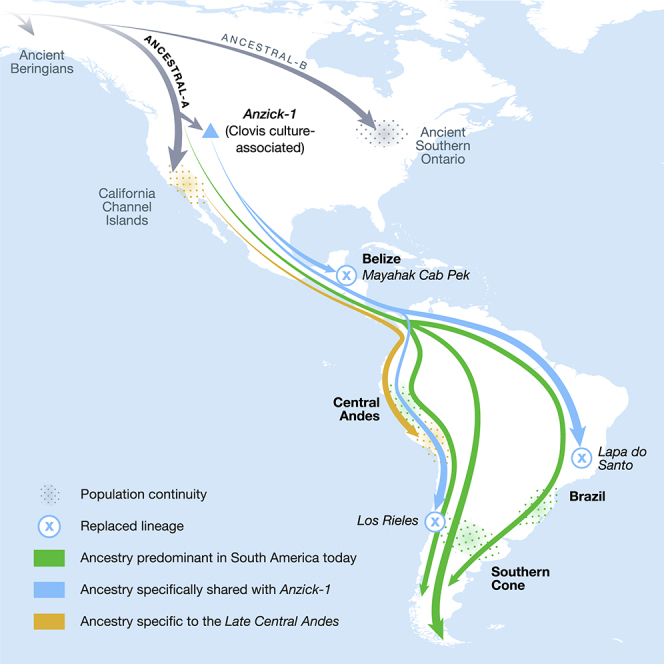

## Teaser

A large-scale analysis of ancient genomes from Central and South America yields insights into the peopling of the Americas, including four southward population spreads and notable population continuity in much of South America after arrival.

## Introduction

Genetic studies of present-day and ancient Native Americans have revealed that the great majority of ancestry in indigenous people in non-Arctic America derives from a homogeneous ancestral population. This population was inferred to have diversified 17,500–14,600 calendar years before present (BP) (Moreno-Mayar et al., 2018a) into two branches that have been called “*Southern Native American*” or “*Ancestral A*” (*ANC-A*) and “*Northern Native American*” or “*Ancestral B*” (*ANC-B*) (Moreno-Mayar et al., 2018a, Raghavan et al., 2015, Rasmussen et al., 2014, Reich et al., 2012, Scheib et al., 2018). An individual dating to ∼12,900–12,700 BP from the Anzick site in Montana and associated with the Clovis culture was on the *ANC-A* lineage, which is also heavily represented in present-day Central and South Americans and in ancient Californians. In contrast, *ANC-B* ancestry is heavily represented in eastern North Americans and in ancient people from southwest Ontario (Scheib et al., 2018). The original studies that documented these two deep lineages fit models in which Central and South Americans were of entirely *ANC-A* ancestry (Rasmussen et al., 2014, Reich et al., 2012). However, Scheib et al. (2018) suggested that all Central and South Americans harbor substantial proportions of both ancestries (at least ∼30% of each).

Recent analyses have also shown that some groups in Brazil share more alleles with Australasians (indigenous New Guineans, Australians, and Andaman Islanders) (Raghavan et al., 2015, Skoglund et al., 2015) and an ∼40,000 BP individual from northern China (Yang et al., 2017) than do other Central and South Americans. Such patterns suggest that these groups do not entirely descend from a single homogeneous population and instead derive from a mixture of populations, one of which, *Population Y*, bore a distinctive affinity to Australasians. Notably, our study includes data from individuals such as those from the Lapa do Santo site who have a cranial morphology known as “Paleoamerican,” argued to indicate two distinct New-World-founding populations (von Cramon-Taubadel et al., 2017). Here, we test directly the hypothesis that a Paleoamerican cranial morphology was associated with a lineage distinct from the one that contributed to other Native Americans (whether the proposed *Population Y* or another).

**Figure 1:**
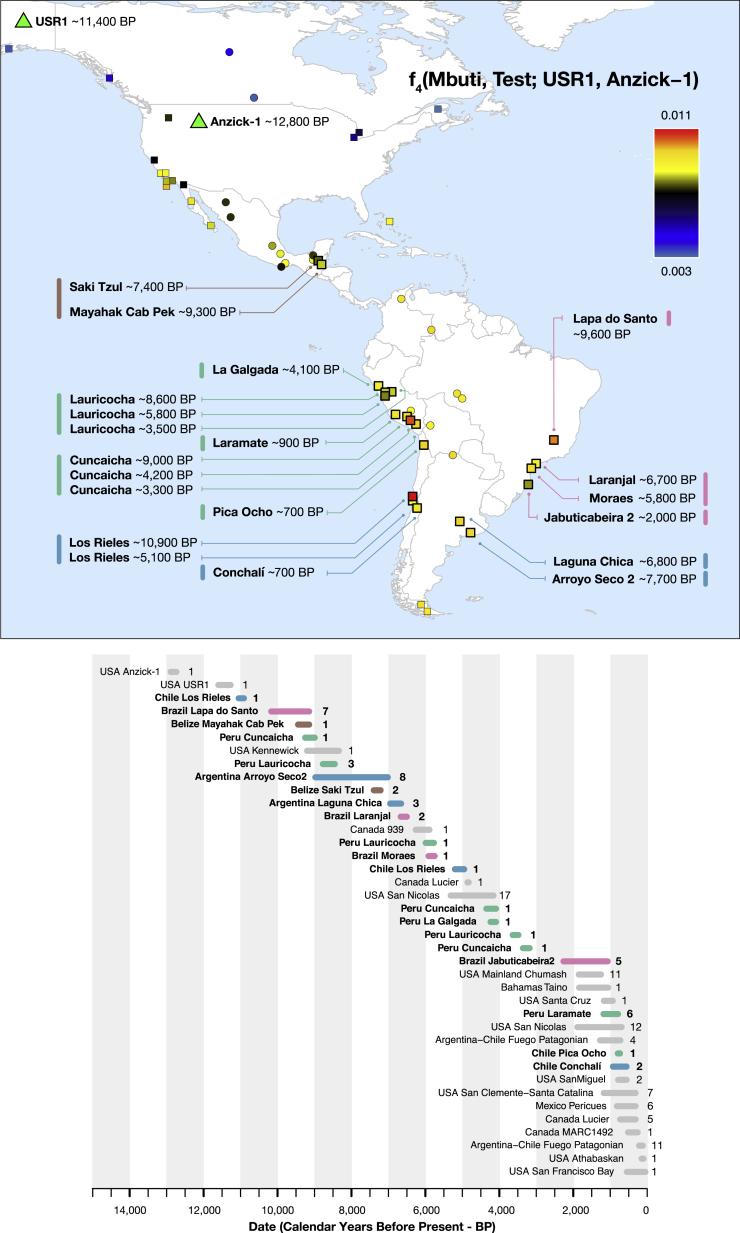
Geographic Locations and Time Ranges (Top) Color coding is based on the value of *f*_*4*_(*Mbuti*, *Test*; *USR1*, *Anzick-1*), which measures the degree of allele sharing of each *Test* population with *Anzick-1* compared to the Ancient Beringian *USR1* (the latter two plotted as green triangles). All values and standard errors are listed in Table S4. Present-day individuals are circles and ancient individuals are squares (the newly reported individuals are indicated with a thick black outline). (Bottom) We show previously published (gray) and newly reported ancient data. Magenta, Brazil; brown, Belize; green, Peru/northern Chile; blue, Southern Cone. The numbers give sample size in each grouping. See also Table S3.

Prior to the present study, published data from Central and South America older than the last millennium was limited to two low coverage genomes (Raghavan et al., 2015). Here, we report genome-wide data from 49 individuals from Belize, Brazil, Peru, and the Southern Cone (Chile and Argentina), 41 older than 1,000 years, with each time transect starting between 10,900–8,600 BP (Figure 1; Table S3). To obtain these data, we worked with government agencies and indigenous peoples to identify samples, prepared powder from skeletal material, extracted DNA (Dabney et al., 2013), and generated single and double stranded DNA libraries, most of which we treated with the enzyme uracil-DNA glycosylase (UDG) to reduce characteristic errors of ancient DNA (Gansauge and Meyer, 2013, Rohland et al., 2015). We enriched for mtDNA and ∼1.2 million SNPs (Fu et al., 2015) and sequenced the enriched libraries on Illumina instruments (Table S3; STAR Methods). We combined ancient and present-day data to study genetic changes over the last 11,000 years.

### Ethics Statement

Genetic studies of human history shed light on how ancient and present-day people are biologically related, and it is therefore important to be attentive not just to scientific issues but also to perspectives of indigenous communities when carrying out this work (Bardill et al., 2018). We took a case-by-case approach in each region we studied. In Peru and in some other countries in Central and South America, there is a strong tradition of indigenism in state policy, and governmental officials are recognized as representatives of indigenous perspectives (Herrera, 2011, Silverman, 2006) (Ley General del Patrimonio Cultural de la Nación [Law No. 28296]). We therefore consulted with provincial and state-based offices of the Ministry of Culture to obtain permission for analysis and also incorporated feedback from local community archaeologists to represent indigenous perspectives; permission for sampling was obtained under Resolución Directoral Nacional No. 1346, 545-2011, and RDN No. 092-2016. In Brazil, we obtained research permits from IPHAN (the National Institute of Historical and Artistic Heritage). In Chile and Argentina, in addition to obtaining permits from the local heritage institutions, we sought to determine if any local indigenous group considered the skeletons we analyzed to be ancestors. For most samples, no indigenous community lived near the sites or indicated a connection to the analyzed skeletons, with the exception of a community living near the site of Laguna Chica in Argentina, which approved the study after consultation and participated in the rescue excavation. In Belize, we obtained permission from the National Institute of Culture and History and the Institute of Archaeology, the legal entities responsible for issuing research permits, and we carried out public consultation with local collaborators and communities (see the archaeological site information section in the STAR Methods for additional details).

## Results and Discussion

### Authenticity of Ancient DNA

We evaluated the authenticity of the isolated DNA based on its harboring: (1) characteristic cytosine-to-thymine mismatches to the reference genome at the ends of the sequenced fragments, (2) point estimates of contamination in mtDNA below 5% (Renaud et al., 2015), (3) point estimates of X chromosome contamination in males below 3% (Korneliussen et al., 2014), and (4) point estimates of genome-wide contamination below 5% (N.N., Éadaoin Harney, S.M., N.P., and D.R., unpublished data). We removed from analysis two individuals that we genetically determined to be first degree relatives of other individuals with higher DNA yields within the dataset but fully report the data for both here (Table S3; STAR Methods).

### Long-Standing Population Continuity in Multiple Regions of South America

We grouped ancient individuals by location, date range, and genetic similarity, for the most part using italicized labels like *Argentina_ArroyoSeco2_7700BP* (“country” followed by “site” followed by a “date” that for us is the average of the midpoint of the date ranges for the individuals in the grouping rounded to the nearest hundred) (Eisenmann et al., 2018). These groupings sometimes span an extensive period of time; for example, the eight Arroyo Seco 2 date estimates range from 8,570 to 7,160 BP. For some analyses, we also lumped individuals into larger clusters, for example grouping individuals from the Andes before and after ∼4,200 BP into “*Early Andes*” and “*Late Central Andes*” based on qualitatively different affinities to other individuals in the dataset (STAR Methods).

To obtain an understanding of how the ancient individuals relate to present-day ones, we computed *f*_*3*_- and *f*_*4*_-statistics, which estimate allele sharing between samples in a way that is unbiased by population-specific drift (Patterson et al., 2012).

**Figure 2:**
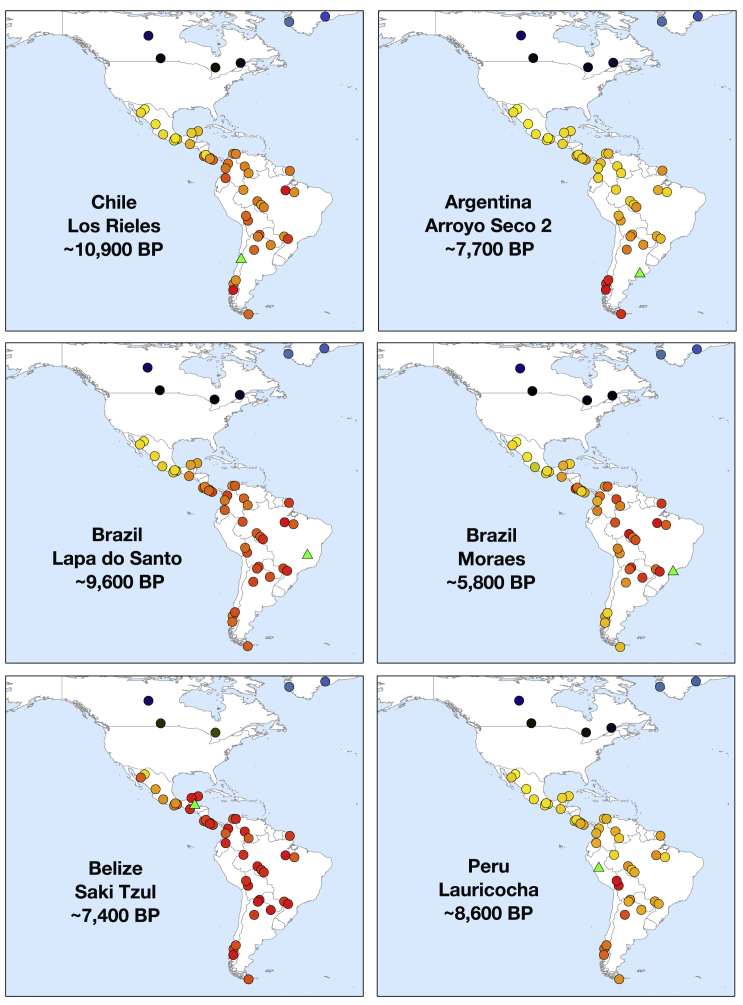
Relatedness of Ancient to Present-Day People Allele sharing statistics of the form f_3_(*Mbuti*; *Test*, *Ancient*), where the “Ancient” individuals represented by a green triangle are *Chile_LosRieles_10900BP*, *Argentina_ArroyoSeco2_7700BP*, *Brazil_LapaDoSanto_9600BP*, *Moraes_Brazil_5800BP*, *Belize_SakiTzul_7400BP*, and *Peru_Lauricocha_8600BP*. The heatmap shows the degree of allele sharing, with red indicating most sharing; yellow, intermediate; and blue, least. See also Figures S2 and S3 and Table S1.

**Figure S1:**
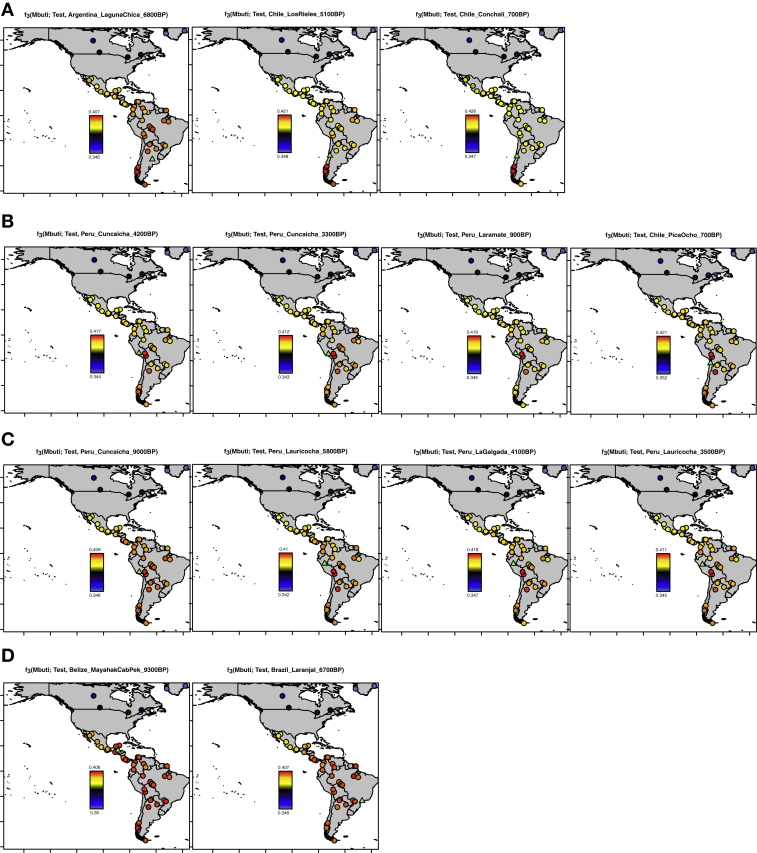
Relatedness of Ancient to Present-Day Individuals, Related to Figure 2 Outgroup *f*_*3*_-statistics of the form *f*_*3*_*(Mbuti; Test, present day Native American*), where *Test* is an ancient individual in Figure 2. (A) Southern Cone (Chile and Argentina) individuals. (B) *Late Central Andes* individuals. (C) *Early Andes* individuals. (D) Brazil and Belize individuals.

The oldest individuals in the dataset show little specific allele sharing with present-day people. For example, a ∼10,900 BP individual from Chile (from the site of Los Rieles) shows only slight excess affinity to later Southern Cone individuals. In Belize, individuals from two sites dating to ∼9,300 and ∼7,400 BP (Mayahak Cab Pek and Saki Tzul) do not share significantly more alleles with present-day people from the region near Belize than they do with present-day groups elsewhere in Central and South America. In Brazil, genetic data from sites dating to ∼9,600 BP (Lapa do Santo) and ∼6,700 BP (Laranjal) show no distinctive shared ancestry with present-day Brazilians (Figures 2 and S1; Table S1), although the Laranjal individuals do show potential evidence of shared ancestry with a ∼5,800 BP individual from Moraes (Table S4), confirmed by the statistic *f*_*4*_*(Mbuti*, *Brazil_Laranjal_6700BP*; *Brazil_LapaDoSanto_9600BP*, *Brazil_Moraes_5800BP*), which is Z = 7.7 standard errors from zero.

**Figure S2:**
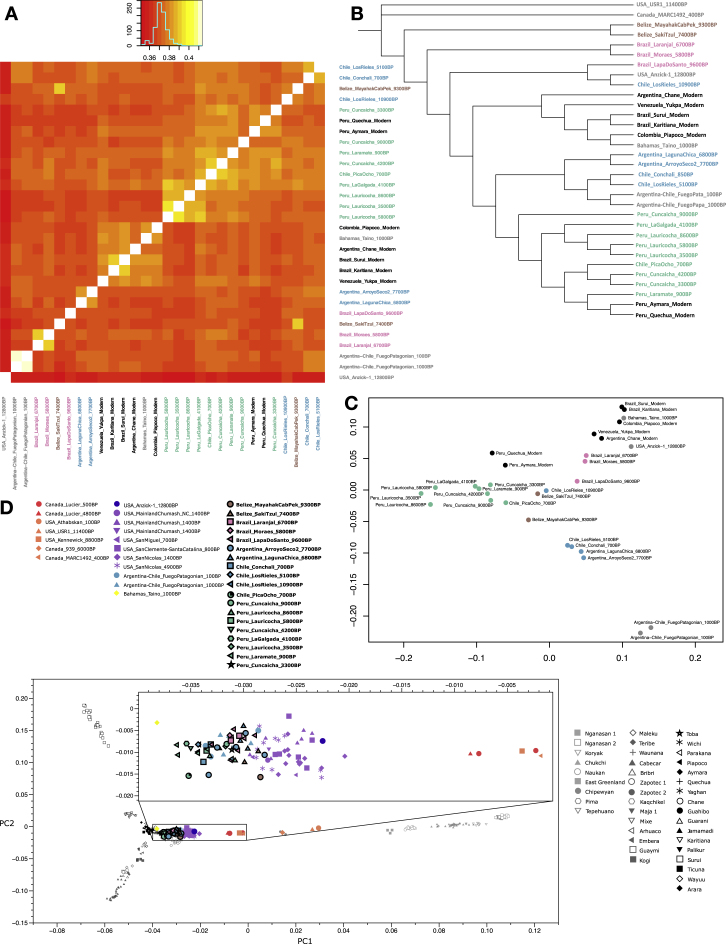
Correlation of Genetics and Geography, Related to Figures 2 and S1 Outgroup *f*_*3*_-statistics of the form *f*_*3*_*(Mbuti;* America_1_, America_2_) for both ancient and present-day Americ an groups for all individuals with over 100,000 SNPs of coverage. (A) Heatmap of the matrix of statistics. (B) Neighbor joining tree of the matrix of inverted statistics (distances = 1/outgroup *f*_*3*_-statistic). (C) MDS plot of the matrix of 1-outgroup *f*_*3*_-statistic. (D) PCA of ancient individuals projected onto present day variation. PCA built with the “unadmixed unmasked” version of the Illumina dataset (STAR Methods); newly reported ancient individuals are projected in black outline.

**Figure S3:**
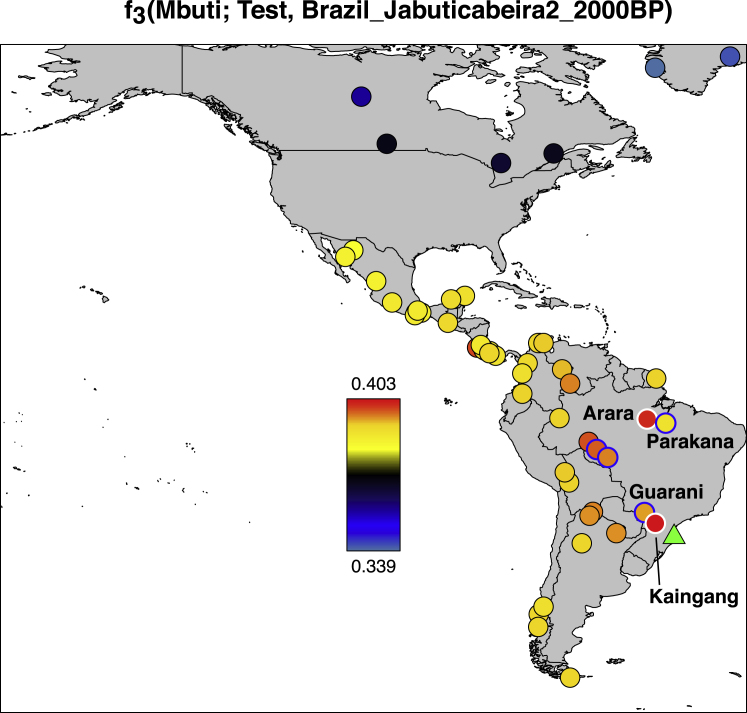
Relatedness of Jabuticabeira 2 Individuals to Present-Day Groups, Related to Figure 2 and Table S1 Outgroup *f*_*3*_-statistics of the form *f*_*3*_*(Mbuti; Brazil_Jabuticabeira2_2000BP, present day Native American*). We marked with blue outline groups that speak Tupi-Guarani languages (*Karitiana*, *Surui*, *Guarani* and *Parakana*) and with a white outline groups that speak Ge languages (*Kaingang*) and Carib languages (*Arara*), and find that the latter two have a specific affinity to *Brazil_Jabuticabeira2_2000BP*. Archeological site location is indicated with a green triangle on each map.

We detect long-standing continuity between ancient and present-day Native Americans in each of the regions of South America we analyzed beginning at least ∼5,800 BP, a pattern that is evident in heatmaps, neighbor-joining trees, and multi-dimensional scaling plots computed on outgroup-*f*_*3*_ statistics (Figures 2, S1, and S2; Table S1). In Peru, the most ancient individuals dating up to ∼9,000 BP from Cuncaicha and Lauricocha share alleles at the highest rate with present-day indigenous groups living in the Central Andes (Lindo et al., 2018, Llamas et al., 2016). Individuals dating up to ∼8,600 BP from Arroyo Seco 2 and Laguna Chica also show the strongest allele sharing with some present-day indigenous people in the Southern Cone. In Brazil, the evidence of continuity with present-day indigenous people begins with the Moraes individual at ∼5,800 BP. A striking pattern of continuity with present-day people is also observed in the ∼2,000 BP Jabuticabeira 2 individuals who were part of the Sambaqui shell-mound building tradition that was spread along the south Brazilian coast from around 8,000–1,000 BP. The Jabuticabeira 2 individuals share significantly more alleles with some Ge-speaking groups than they do with some Tupi-Guarani speaking groups who have been predominant on the coast during the post-Colonial period (Figure S3; Table S1). This supports the theory of shared ancestry between the makers of the Sambaqui culture and the speakers of proto-Ge who are hypothesized to have lived in the region ∼2,000 BP (Iriarte et al., 2017). These findings also support the theory of coastal replacement of Ge speakers by Tupi-Guarani speakers after ∼1,000 BP (Hubbe et al., 2009) (STAR Methods).

### Evidence for at Least Four Genetic Exchanges between South America and Other Regions

Figure 1 plots the excess rate of allele sharing of ancient Central and South Americans with the ∼12,800 BP *Anzick-1* individual from Montana compared to the ∼11,500 BP *USR1* individual from Alaska, an Ancient Beringian who derives from a lineage that split from the one leading to all other known Native Americans before they separated from each other (Moreno-Mayar et al., 2018a) (Table S4). The distribution of this statistic *f*_*4*_*(Mbuti*, *Test*; *USR1*, *Anzick-1*) confirms previous findings that *Anzick-1* relatedness is greatest in Central and South Americans and lowest in North American groups (Table S4) (Rasmussen et al., 2014), with the exception of the California Channel Islands, where the earliest individuals from San Nicolas Island around 4,900 BP show some of the highest *Anzick-1* relatedness, consistent with an early spread of *Anzick-1*-related people to these islands followed by local isolation (Scheib et al., 2018) (Figure S2D).

**Figure S4:**
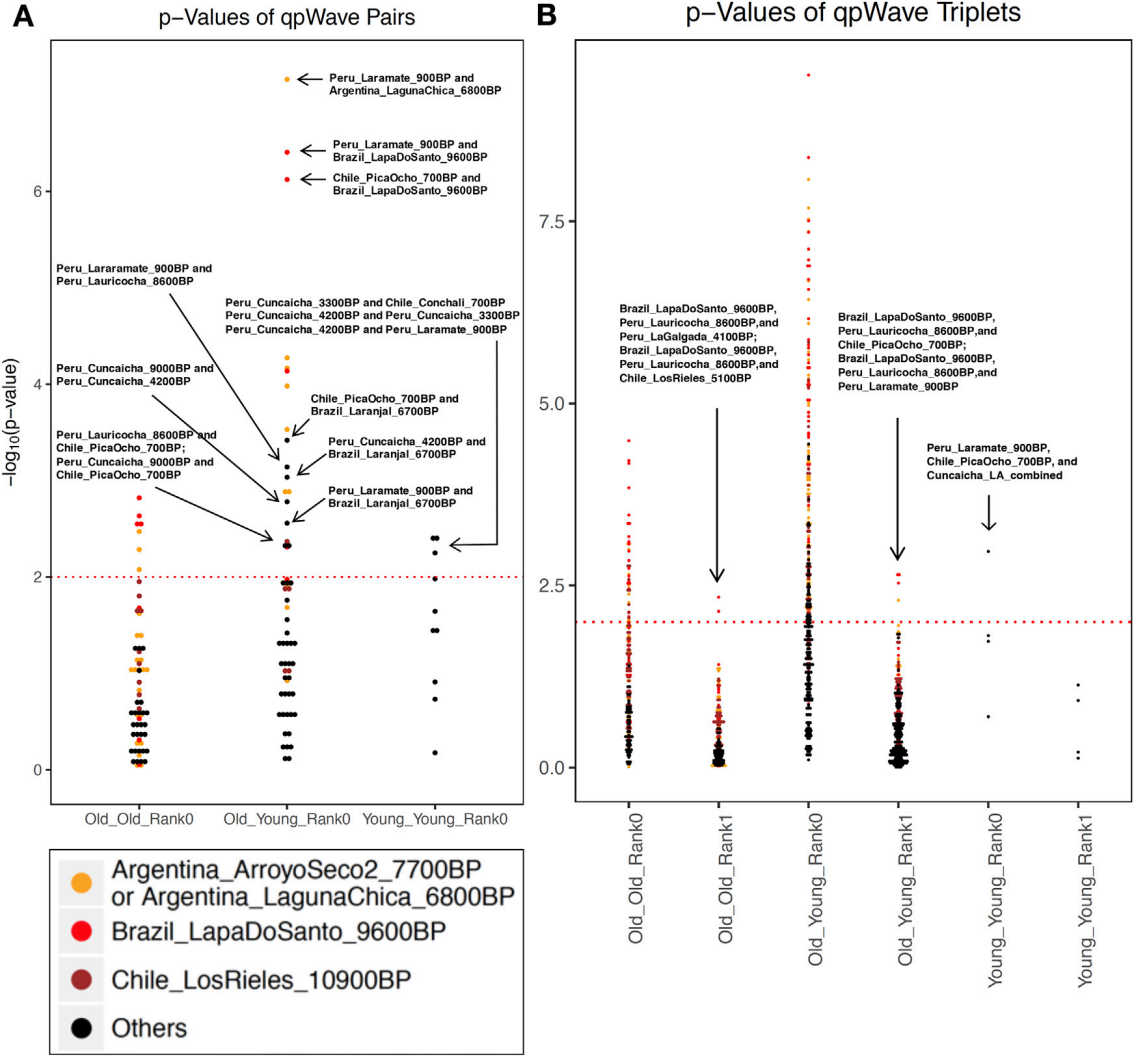
Minimum Number of Ancestral Sources, Related to Figures 4 and 5 *qpWave* analyses with all (A) pairs and (B) triplets of South American groups as “left” populations (related to Table S5, which also shows quadruplet and quintuplet statistics). Southern Cone, Belize, Brazil, and *Early Andes* individuals are labeled “Old,” while *Late Central Andes* individuals are labeled “Young.” Only individuals with over 100,000 SNPs covered were used for this analysis. The colors of the dots indicate whether the combination includes *Brazil_LapaDoSanto_9600BP* (red), *Chile_LosRieles_10900BP* (brown) or *Argentina_ArroyoSeco2_7700BP* or *Argentina_LagunaChica_6800BP* (orange). *Cuncaicha_LA_combined* refers to a combination of *Peru_Cuncaicha_4200BP* and *Peru_Cuncaicha_3300BP*. Rank 0 and 1 refers to a model in which all populations in the analysis fit as derived from one or two ancestral populations, respectively, relative to the outgroups (rejection of these ranks means that additional waves of ancestry are required to model the populations).

More careful examination reveals significant ancestry variability in the ancient South Americans. The ∼10,900 BP Los Rieles individual from Chile, the ∼9,600 BP individuals from Lapa do Santo in Brazil, and individuals from southern Peru and northern Chile dating to ∼4,200 BP and later (“Late Central Andes” from Cuncaicha, Laramate and Pica Ocho), share more alleles with *Anzick-1* than do other South Americans (Figure 1; Table S4). Many of these signals of asymmetrical relationship to *Anzick-1* are significant as assessed by statistics of the form *f*_*4*_*(Mbuti*, *Anzick-1*; *Test*_*1*_, *Test*_*2*_): *Z* score for deviation from zero as high as 3.4 for the (*Test*_*1*_, *Test*_*2*_) pair (*Early Andes*, *Chile_LosRieles_10900BP*), 3.1 for the pair (*Early Andes, Brazil_LapaDoSanto_9600BP*), and 3.0 for the pair *(Early Andes*, *Late Central Andes*) (Table S2). We confirmed these findings using *qpWave* (Reich et al., 2012), which evaluates the minimum number of sources of ancestry that must have contributed to a test set of groups relative to a set of outgroups (STAR Methods). We tested all possible pairs of populations and found that none of the three combinations are consistent with being derived from a homogeneous ancestral population: p = 0.0023 for (*Early Andes*, *Brazil_LapaDoSanto_9600BP*), p = 0.0007 for (*Early Andes*, *Late Central Andes*), and p = 0.0000004 for (*Brazil_LapaDoSanto_9600BP*, *Late Central Andes*). We obtained qualitatively similar results replacing *Brazil_LapaDoSanto_9600BP* with *Chile_LosRieles_10900BP* (Figure S4; Table S5). We also obtained similar results for subsets of individuals in each group. Our power to reject models of just two sources of ancestry for the ancient South American individuals depends critically on the use of *Anzick-1* as an outgroup, as when we remove this individual from the outgroup set there is no evidence of a third source of ancestry contributing to *Brazil_LapaDoSanto_9600BP* (p = 0.11) or *Chile_LosRieles_10900BP* (p = 0.35). It also depends critically on the use of California Channel Islands individuals, as when we remove them as outgroups there is no evidence for a third source of ancestry contributing to *Late Central Andes* groups (p = 0.12).

The fact that the three pairs each require two different sources of ancestry in order to produce a model fit could mean that they descend from a total of three (or more) distinct sources of ancestry differentially related to groups outside South America or alternatively that they are mixtures in different proportions of only two sources. To distinguish these possibilities, we used *qpWave*’s ability to test for consistency with the hypothesis that sets of three populations (*Test*_*1*_, *Test*_*2*_, *Test*_*3*_) derive from just two populations relative to the same set of outgroups. *qpWave* rejects the hypothesis of two sources (p = 0.0022), a result that is unlikely to be due to backflow from South America into Central America as the signal persists when we remove present-day Mexicans from the outgroup set (p = 0.001) (Table S5). Further evidence for the robustness of the finding of three source populations comes from the fact that the signal remains significant when we restrict to transversion polymorphisms that are not affected by cytosine-to-thymine errors (p = 0.01). We caution that we did not find significant signals of ancestry heterogeneity relative to North American outgroups when repeating the *qpWave* tests on pairs of present-day populations. We speculate that this may reflect more recent homogenization leading to variation in ancestry proportions too subtle for our methods to detect.

When we add present-day *Surui* individuals into the analysis, there is evidence for a fourth source of ancestry (p = 0.03) (Table S5), likely reflecting the same signal that led to finding “*Population Y*” ancestry in this group (Raghavan et al., 2015, Skoglund et al., 2015).

### Modeling the Deep History of Central and South America

We modeled the relationships among diverse ancient Americans using *qpGraph,* which evaluates whether a model of population splitting and admixture is consistent with all *f-*statistics relating pairs, triples, and quadruples of groups (Patterson et al., 2012).

**Figure 3:**
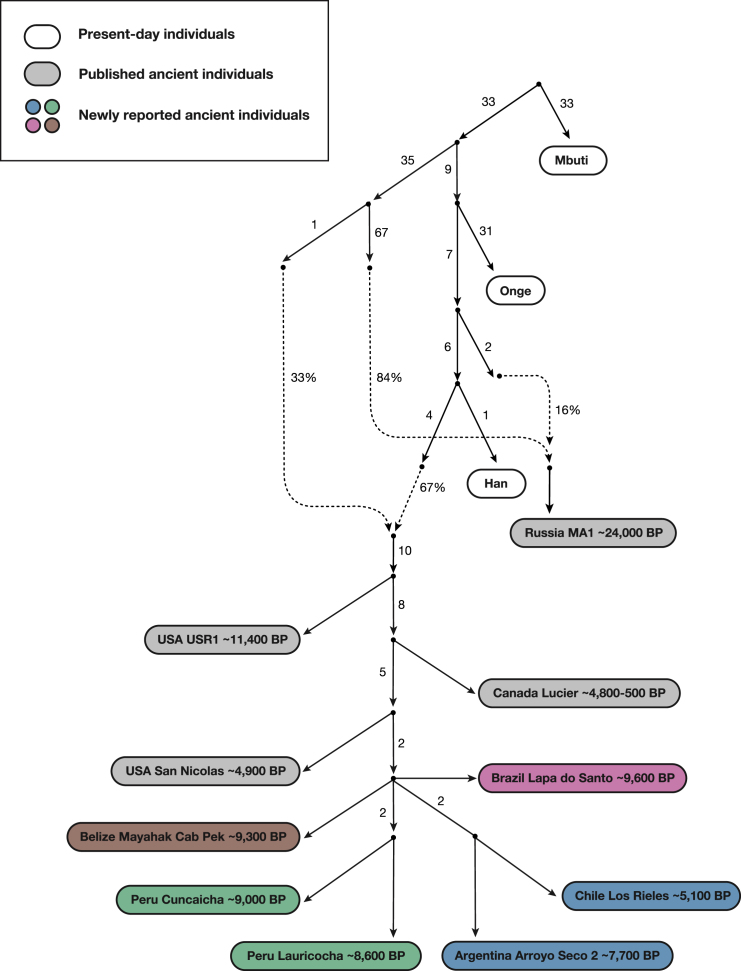
Skeleton Model that Fits the Data with Minimal Admixture This graph models nine of the ancient North, Central, and South American groups without admixture (branch lengths are in units of F_ST_ × 1,000). The maximum deviation between observed and expected *f*-statistics is *Z* = 2.9 (*Z* = 3.1 when restricting to transversions). Drift lengths in the terminal edges are unlabeled as randomly sampling an allele to represent each individual makes them artifactually long. See also Figure S6.

**Figure S5:**
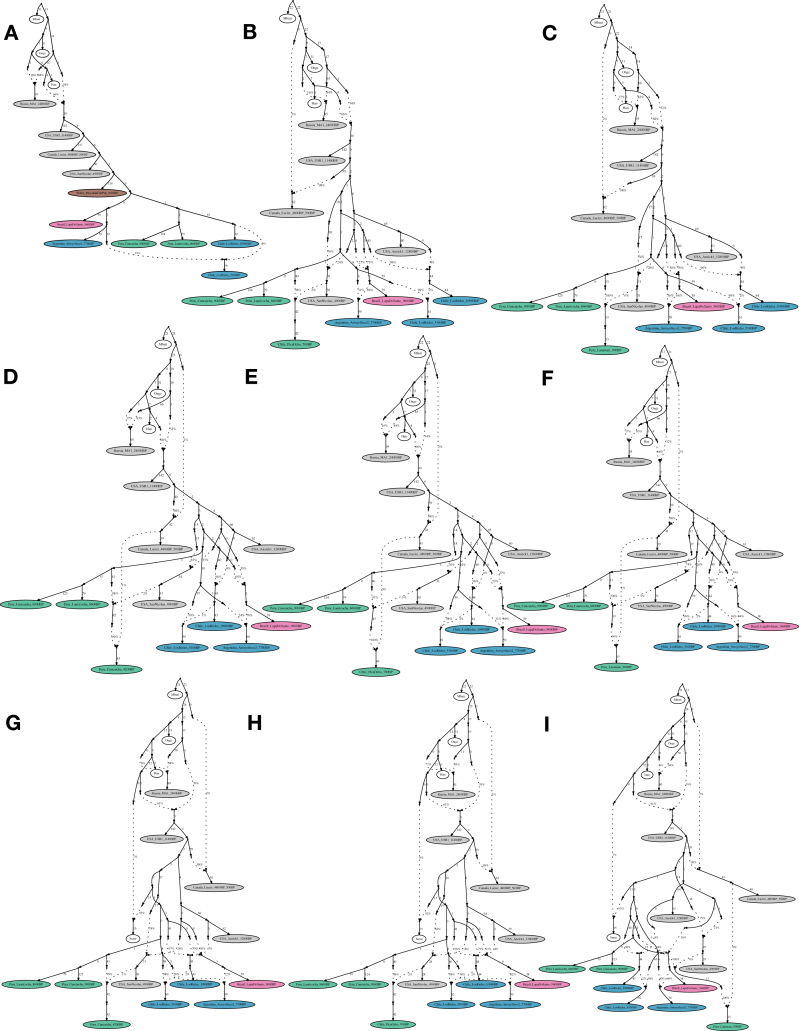
Alternative Admixture Graphs, Related to Figures 4 and 5 (A) Admixture graph in the same format as Figure 3 except with *Chile_LosRieles_10900BP* added. The maximum Z-score is 3.2 (we give in parentheses the value when restricting to transversions, here 3.1). The significant shared ancestry between the two Los Rieles individuals is indicated by statistics such as *f4(Mbuti, Chile_LosRieles_10900BP; Argentina_ArroyoSeco2_7700BP, Chile_LosRieles_5100BP)*, which gives Z = 2.8. The following graphs have the same format of Figure 4 but (B) with *Chile_PicaOcho_700BP* instead of *Peru_Cuncaicha_4200BP*. The maximum Z-score is 3.4 (4.7, a signal of poor fit that may be an artifact of extremely low coverage of *Chile_PicaOcho_700BP* when restricting to transversions). (C) with *Peru_Laramate_900BP* instead of *Peru_Cuncaicha_4200BP*, which gives a maximum Z-score of 3.6 (3.5). Admixture graphs with an extra added admixture edge of *ANC-B* (D) for *Peru_Cuncaicha_4200BP* (maximum Z-score is 3.3 (3.0)), (E) for *Chile_PicaOcho_700BP* (maximum Z-score is 3.4 (4.8)), and (F) for *Peru_Laramate_900BP* (maximum Z-score is 3.5 (3.5)). Admixture graph including *Surui*, which shows the necessity of additional East Asian-related ancestry into (G) *Peru_Cuncaicha_4200BP* (maximum Z-score is 4.2 (3.7)). (H) *Chile_PicaOcho_700BP* (maximum Z-score is 4.0 (4.6)). (I) *Peru_Laramate_900BP* (maximum Z-score is 4.1 (3.5)).

**Figure S6:**
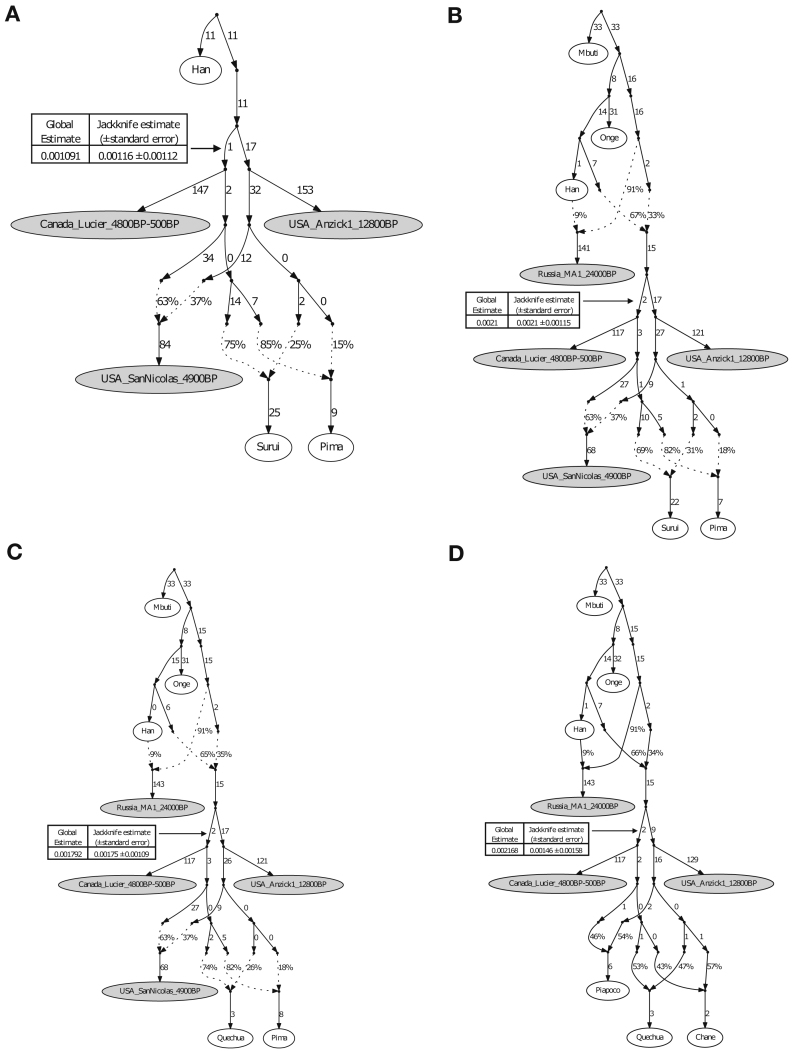
No Evidence for Widespread *ANC-B* Ancestry in the Americas, Related to Figures 3–5 (A) Admixture graph in the same format as for Figure 2A of Scheib et al. (2018) (we left out *USA_SanNicolas_1400BP* (*LSN* in Scheib et al., 2018) from our modeling due to its known relationship with *Pima*, which would lead to higher maximum Z-scores of the later graphs). This graph has a maximum Z-score of 1.1 for mismatch between observed and expected *f*-statistics. (B-D) Admixture graphs in the same format as for A but with additional non-American populations with known relationships to American ones added as outgroups. B shows a poor fit (maximum Z-score = 4.8), likely due to lack of modeling of the “*Population Y*” signal. C and D have reasonable fits (maximum Z-scores = 3.4 and 3.0, respectively), but the genetic drift on the edge leading to *Canada_Lucier_4800BP-500BP* (*ASO* in Scheib et al., 2018) in all cases is not significantly different from zero when computing jackknife estimates by resampling over 100 contiguous blocks. Thus, the ancestry on the *Canada_Lucier_4800BP-500BP* branch that mixes into the South American groups does not share a significant amount of drift with *Canada_Lucier_4800BP-500BP* (see STAR Methods for more details).

We were able to fit genome-wide data from nine ancient North, Central and South American groups (not including *Anzick-1*) as a star-like radiation from a single source population with negligible admixture between the *ANC-A* and *ANC-B* lineages after their initial bifurcation (maximum |*Z*| score for a difference between the observed and expected statistics of 2.9 [Figure 3] and 3.2 [Figure S5A]; we represent *ANC-B* by the Ancient Southern Ontario population *Canada_Lucier_4800BP-500BP*). This model is not what would be expected based on the claim of a recent study (Scheib et al., 2018) that major *ANC-A*/*ANC-B* admixture (at least ∼30% of each) is necessary to model Central and South Americans. While we confirmed that the model proposed in Scheib et al. (2018) fits the data when restricting to a subset of the populations they analyzed, when we added into the model non-American populations with previously established relationships to Native Americans, the model failed (STAR Methods). To more directly explore whether there is evidence of widespread *ANC-B* ancestry in South America, we tested whether *Canada_Lucier_4800BP-500BP* shares more alleles with a range of Central and South American *Test* populations than with *Anzick-1,* but find no evidence for a statistically significant skew (Table S4). Indeed, the supplementary materials of the previously reported study (Figure S13 of Scheib et al., 2018) show that a model such as the one we favor—without widespread *ANC-B* admixture in South America—fits the data with no differences between observed and expected *f*-statistics greater than *Z* > 2. We also find that when we explicitly model *ANC-B* admixture into the ancestors of South Americans, the inferred genetic drift specific to *Canada_Lucier_4800BP-500BP* is not significantly different from 0, providing evidence against specific affinity to *ANC-B* in South Americans (Figure S6; STAR Methods).

**Figure 4:**
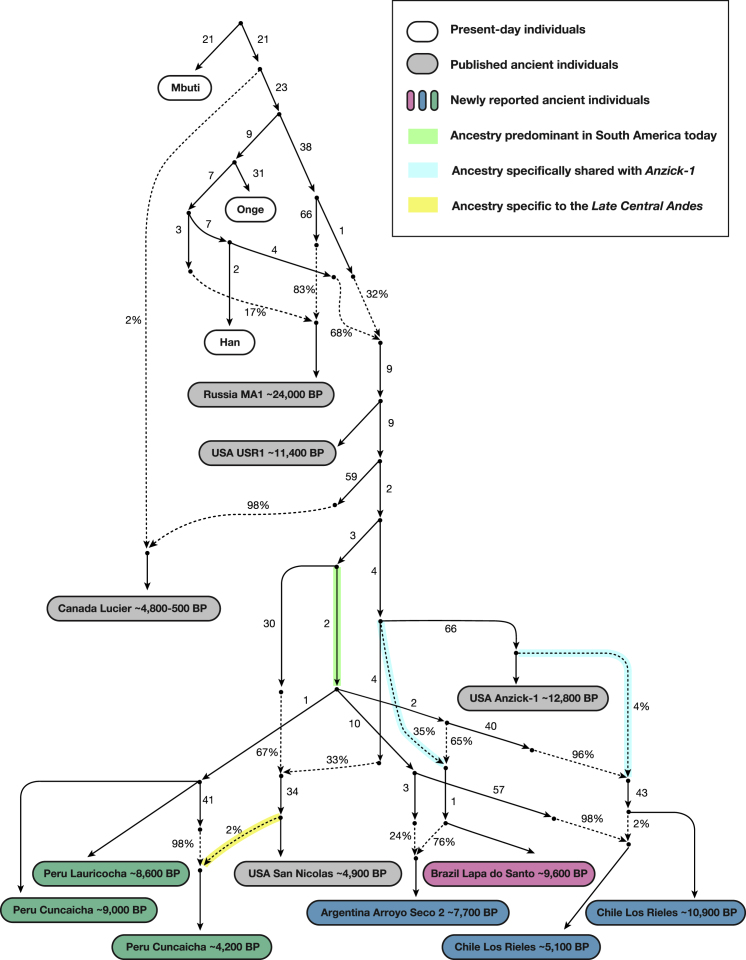
Adding in the ∼12,800 BP Anzick-1 and ∼10,900 BP Los Rieles We used Figure 3 that models all analyzed Native Americans as unadmixed as a framework graph (excluding *Belize_MayahakCabPek_9300BP* because of relatively low coverage). We then added in *Anzick-1 and Chile_LosRieles_10900BP*. This model specifies three sources of North American related ancestry in South America, indicated by color-coding (*Population Y* ancestry is not included but Figures S5B–S5I show related fits some of which do include it). The maximum deviation between observed and expected *f*-statistics is *Z* = 3.4 (*Z* = 3.0 when restricting to transversions). The inferred 2% West Eurasian admixture into *Canada_Lucier_4800BP-500BP* is most likely explained by contamination in these samples by people of European ancestry. See also Figure S6 and Tables S4 and S5.

**Figure 5:**
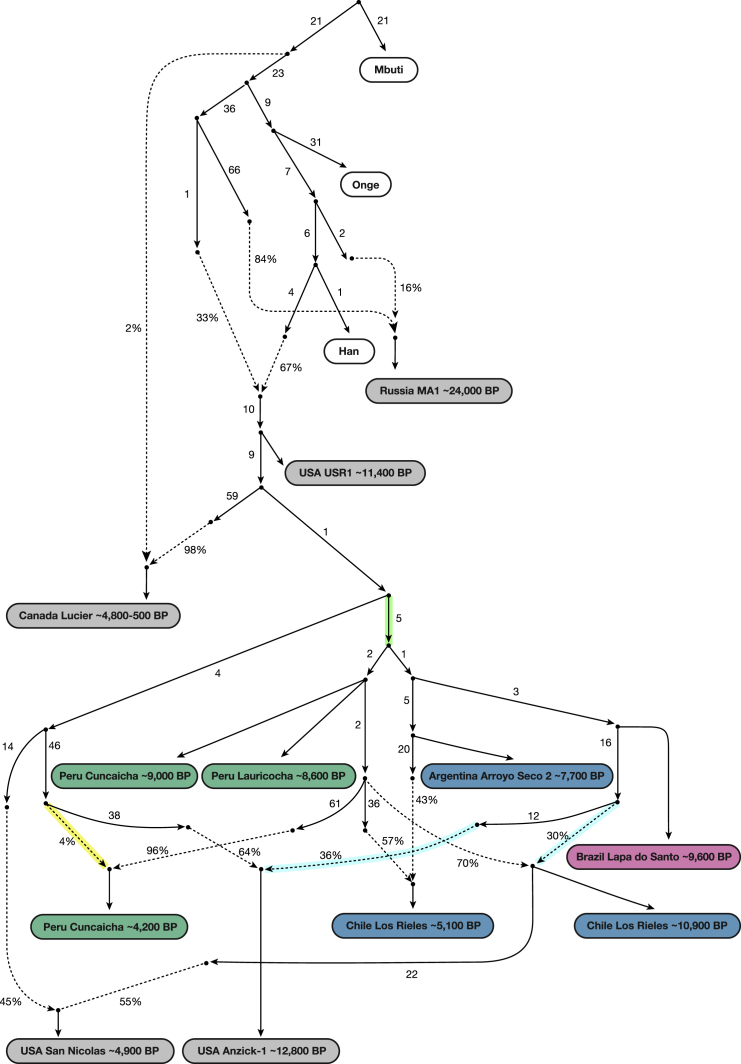
An Alternative Fitting Admixture Graph Obtained by a Semi-automated Method We also applied a semi-automated approach that aims to fit population relationships while minimizing the number of admixture events (STAR Methods) (Lazaridis et al., 2018). This is less plausible than Figure 4 on archaeological grounds, but it has a lower maximum *Z* score for the same number of admixture edges (*Z* = 2.9 for all sites, *Z* = 2.9 when restricting to transversions). Like Figure 4, this model specifies a minimum of three genetic exchanges between North and South America, indicated here by color-coding (please see Figure 4 color legend). See also Figure S6 and Table S5.

To fit the *Anzick-1* genome associated with the Clovis culture into the admixture graph, we needed to specify additional admixture events. We identified a range of fits for the data. Figure 4 shows the result of manually exploring models guided by common sense principles (geography, time, and archaeology) as well as the genetic data. Figure 5 shows a model obtained by a semi-automated procedure constrained only by the fit to the genetic data (Lazaridis et al., 2018). The most important difference between the two models concerns the question of how the Clovis culture associated *Anzick-1* genome relates to ancient Central and South Americans. Figure 4, which models the lineage leading to *Anzick-1* as unadmixed, seems most plausible because it is natural to expect that the oldest individuals will be least admixed, and because it is simple to explain this model via North-to-South spreads. Figure 5 models some of the ancestry of the Clovis associated genome as deriving from within the radiation of lineages represented in South America, which if true would require a more complex history.

We highlight four points of agreement between the two admixture graphs.

First, both graphs imply a minimum of four genetic exchanges between South America and regions outside South America consistent with the *qpWave* results in the previous section. This includes: (1) a primary source of *ANC-A* ancestry in all South Americans; (2) an *ANC-A* lineage with distinct affinity to *Anzick-1* in *Chile_LosRieles_10900BP*, *Brazil_LapaDoSanto_9600BP*, and some early Southern Cone populations; and (3) *ANC-A* ancestry with a distinctive affinity to ancient individuals from the California Channel Islands (*USA_SanNicolas_4900BP*) present in the Central Andes by ∼4,200 BP (Figures S5B and S5C). (4) The final spread of ancestry contributes to present-day Amazonian groups like the *Surui*. In Figures 4 and 5, we do not include the *Surui* but do show such models in Figures S5G–S5I where *Surui* can only be fit by proposing some ancestry differently related to Eurasians than is the case for other Native Americans (as expected if there is *Population Y* ancestry in the *Surui*).

Second, both graphs specify minimal *ANC-B* ancestry in South Americans. While we do find significant allele sharing with a representative *ANC-B* population (*Canada_Lucier_4800BP-500BP*) in people from the Central Andes after ∼4,200 years ago—as reflected in significantly positive (2 < *Z* < 4) statistics of the form *f*_*4*_(*Mbuti*, *Canada_Lucier_4800BP-500BP*; *Brazil_LapaDoSanto_9600BP* or *Brazil_Laranjal_6700BP*, *Late Central Andes or present-day Aymara and Quechua from Peru*) (Tables S2 and S4)—when we fit admixture graph models specifying an *ANC-B* contribution to *Late Central Andes* groups, the *ANC-B* proportion is never more than 2% (Figures S5D–S5F).

Third, both graphs infer little genetic drift separating the lineages leading to the different ancient groups in each major region of South America. This can be seen in our inferred five-way split whose order we cannot resolve involving lineages leading to: (1) the early Belizeans, (2) early Peruvians, (3) early Southern Cone populations, (4) the main lineage leading to *Brazil_LapaDoSanto_9600BP*, and (5) the lineage leading to *Chile_LosRieles_10900BP* (Figure S5A). This suggests rapid human radiation of the main lineage ancestral to later South Americans (Raghavan et al., 2015, Reich et al., 2012).

Fourth, both graphs agree that there is distinctive shared ancestry between the Clovis culture associated *Anzick-*1 and the earliest South American individuals from Lapa do Santo in Brazil and Los Rieles in Chile. We also detect evidence of ancestry related to *Anzick-1* in the oldest Central American genome, as the most ancient individual from Belize has evidence of more *Anzick-1* relatedness than later Belize individuals as reflected in the weakly significant statistic *f*_*4*_(*Mbuti, Anzick-1*; *Belize_SakiTzul_7400BP*, *Belize_MayahakCabPek_9300BP*) (*Z* = 2.1). Taken together, these results support the hypothesis that an expansion of a group associated with the Clovis culture left an impact far beyond the geographic region in which this culture was spread (Fiedel, 2017). At the same time, both classes of models provide evidence against a stronger version of this hypothesis, which is that an expansion of a homogeneous population associated with the Clovis culture was the primary source of the ancestry of later Central and South Americans. Specifically, both models find that the overwhelming majority of the ancestry of most Central and South Americans derives from one or more lineages without the *Anzick-1* affinities present at Lapa do Santo. Thus, a different *ANC-A* lineage from the one represented in *Anzick-1* made the most important contribution to South Americans, and there must have been a population turnover in the mid-Holocene that largely replaced groups such as the ones represented by the ∼10,900 BP individual at Los Rieles in Chile and the ∼9,600 BP individuals at Lapa do Santo in Brazil. This genetic evidence of a major population turnover correlates with the findings from morphological studies of a population turnover in Brazil around this time (Hubbe et al., 2014).

It is tempting to hypothesize that the early branching *ANC-A* lineages that we have shown contributed most of the ancestry of Central and South Americans today—and that harbor no specific *Anzick-1* association—contributed to the people who lived at the site of Monte Verde in southern Chile and whose material artifacts have been dated to a pre-Clovis period at least ∼14,500 BP (Dillehay et al., 2008). However, because all the earliest Central and South American individuals show affinities to *Anzick-1*, our results could also be consistent with a scenario in which nearly all the ancestry of the South American genomes derives from population movements from North America that began no earlier than the Clovis period. In either case, we demonstrate that the non-*Anzick-1* associated ancestry type began to spread in South America by at least ∼9,000 BP, the date of the oldest genomes that have no specific Anzick-1 affinity (from Cuncaicha and Lauricocha in the Central Andes).

### All the Ancient South Americans Descend from the Same Eurasian Source Population

Previous studies have suggested that present-day groups like *Surui* from Amazonia harbor ancestry from a source termed “*Population Y*” (Raghavan et al., 2015, Skoglund et al., 2015), which shared alleles at an elevated rate with Australasian groups (*Onge*, *Papuan*, and *Australians*) as well as the ∼40,000 BP Tianyuan individual from China (Yang et al., 2017). We tested for this signal in the ancient South American individuals with statistics of the form *f*_*4*_(*Mbuti, Australasian*; *X*, *Mixe or ancient South American*), and while we replicated the originally reported signal when *X* was present-day *Karitiana* or *Surui*, we could not detect a signal when *X* was any of the ancient South Americans (Table S6). We also studied the statistic *f*_*4*_(*Mbuti*, *Tianyuan*; *Ancient*_*1*_, *Ancient*_*2*_) to test if any ancient individual is differentially related to Tianyuan (Yang et al., 2017), but no statistic was significant (Table S6). We finally applied *qpWave* to all pairs of South American groups, testing whether they were homogeneously related to a set of diverse non-Native American outgroups (*Mbuti*, *Han*, *Onge*, *French*, and *Papuan*) and found no pair of ancient South Americans that consistently gave significant signals (p < 0.01), as expected if all the ancient South Americans we analyzed derived from the same stem Native American population (Table S6). Our failure to find significant evidence of Australasian or Paleolithic East Asian affinities in any of the ancient Central and South American individuals raises the question of what ancient populations could have contributed the *Population Y* signal in *Surui* and other Amazonian groups and increases the previously small chance that this signal—despite the strong statistical evidence for it—was a false-positive. A priority is to search for the *Population Y* signal in additional ancient genomes.

Our finding of no excess allele sharing with non-Native American populations in the ancient samples is also striking as many of these individuals—including those at Lapa do Santo—have a “Paleoamerican” cranial morphology that has been suggested to be evidence of the spread of a substructured population of at least two different Native American source populations from Asia to the Americas (von Cramon-Taubadel et al., 2017). Our finding that early Holocene individuals with such a morphology are consistent with deriving all their ancestry from the same homogeneous ancestral population as other Native Americans extends the finding of Raghavan et al. (2015) who came to a similar conclusion after analyzing Native Americans inferred to have Paleoamerican morphology who lived within the last millennium.

### Single Locus Analysis

**Figure S7:**
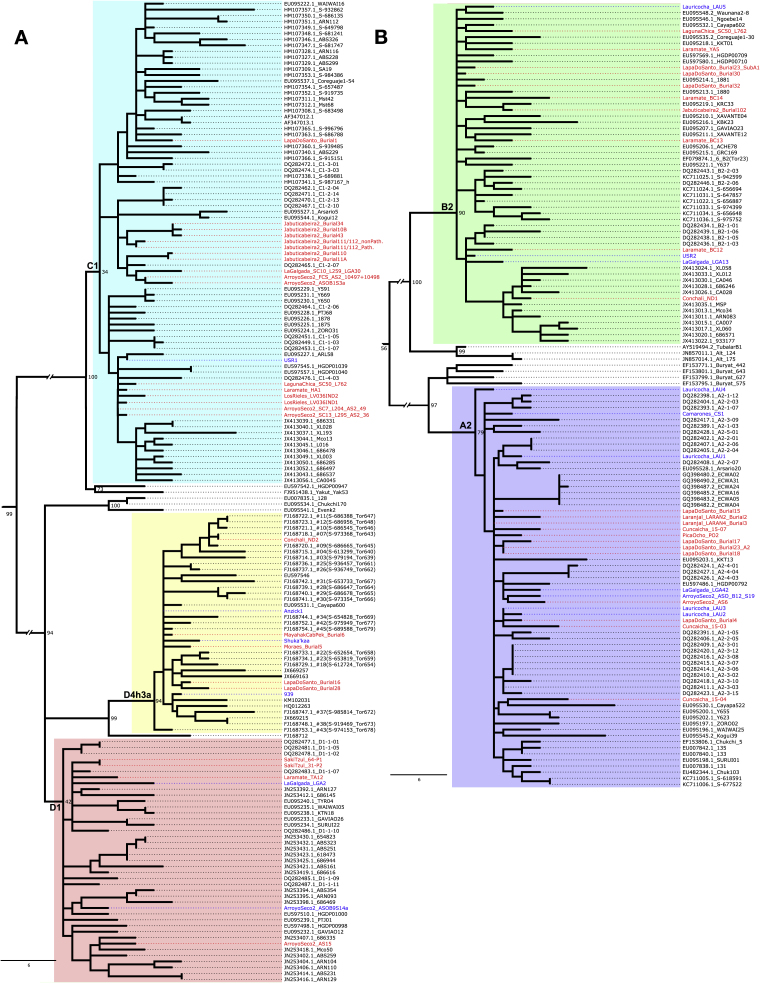
Mitochondrial DNA Phylogeny, Related to Figure 3, Figure 4, Figure 5 (A) Maximum parsimony phylogenetic tree of 65 ancient mtDNA (previously published sequences are in blue font and newly reported sequences are in red font) and 230 modern mtDNA sequences (in black font) built using MEGA6 (Tamura et al., 2013). Related to Table S3. The African mtDNA L haplogroup was used to root the tree (not shown). (A) Tree portion that includes mtDNA haplogroup C1, D4h3a and D1; (B) Tree portion that includes mtDNA haplogroup A2 and B2. The mtDNA sequence of individual *LagunaChica_SC50_L763* (Table S3) is not reported in this tree due to the high proportion of unassigned positions (2444Ns).

The D4h3a mtDNA haplogroup has been hypothesized to be a marker for an early expansion into the Americas along the Pacific coast (Perego et al., 2009). However, its presence in two Lapa do Santo individuals and *Anzick-1* (Rasmussen et al., 2014) makes this hypothesis unlikely (Figure S7; Table S3; STAR Methods).

The patterns we observe on the Y chromosome also force us to revise our understanding of the origins of present-day variation. Our ancient DNA analysis shows that the Q1a2a1b-CTS1780 haplogroup, which is currently rare, was present in a third of the ancient South Americas. In addition, our observation of the currently extremely rare C2b haplogroup at Lapa do Santo disproves the suggestion that it was introduced after 6,000 BP (Roewer et al., 2013).

The patterns of variation at phenotypically significant variants are also notable. Our data show that a variant in *EDAR* that affects tooth shape, hair follicles and thickness, sweat, and mammary gland ductal branching and that occurs at nearly 100% frequency in present day Native Americans and East Asians (Kamberov et al., 2013) was not fixed in *USR1*, *Anzick-1*, a *Brazil_LapaDoSanto_9600BP* individual and a *Brazil_Laranjal_6700BP* individual, all of whom carry the ancestral allele (Table S7). Thus, the derived allele rose in frequency in parallel in both East Asians and in Native Americans. In contrast at *FADS2*, one of the variants at a polymorphism (rs174570) associated with fatty acid desaturase 2 levels is derived in all the ancient individuals, supporting the hypothesis that the selective sweep that drove it to near fixation was complete prior to the peopling of the Americas (Amorim et al., 2017).

## Discussion

Our finding of two previously undocumented genetic exchanges between North and South America has significant implications for models of the peopling of the Americas.

Most important, our discovery that the Clovis-associated *Anzick-1* genome at ∼12,800 BP shares distinctive ancestry with the oldest Chilean, Brazilian, and Belizean individuals supports the hypothesis that an expansion of people who spread the Clovis culture in North America also affected Central and South America, as expected if the spread of the Fishtail Complex in Central and South America and the Clovis Complex in North America were part of the same phenomenon (direct confirmation would require ancient DNA from a Fishtail-context) (Pearson, 2017). However, the fact that the great majority of ancestry of later South Americans lacks specific affinity to *Anzick-1* rules out the hypothesis of a homogeneous founding population. Thus, if Clovis-related expansions were responsible for the peopling of South America, it must have been a complex scenario involving arrival in the Americas of sub-structured lineages with and without specific *Anzick-1* affinity, with the one with *Anzick-1* affinity making a minimal long-term contribution. While we cannot at present determine when the non*-Anzick-*1 associated lineages first arrived in South America, we can place an upper bound on the date of the spread to South America of all the lineages represented in our sampled ancient genomes as all are *ANC-A* and thus must have diversified after the *ANC-A*/*ANC-B* split estimated to have occurred ∼17,500–14,600 BP (Moreno-Mayar et al., 2018a).

A second notable finding of this study is our evidence that the ancient individuals from the California Channel Islands have distinctive and significant allele sharing with groups that became widespread over the Central Andes after ∼4,200 BP. There is no archaeological evidence of large-scale cultural exchange between North and South America around this time, but it is important to recognize that ∼4,200 BP is a minimum date for the exchange between North and South American that drove this pattern; the gene flow itself could have occurred thousands of years before and the ancestry deriving from it could have persisted in a region of South America not yet sampled with ancient DNA. The evidence of an expansion of this ancestry type in the Central Andes by ∼4,200 BP is notable in light of the increasing density of sites in this region at approximately this time, a pattern that is consistent with a demographic expansion of a previously more restricted population (Goldberg et al., 2016).

We conclude by highlighting several limitations of this study. First, all the individuals we newly report have a date less than ∼11,000 BP and thus we could not directly probe the initial movements of people into Central and South America. Second, from the period between 11,000–3,000 BP that includes most of our individuals, we lacked ancient data from Amazonia, northern South America, and the Caribbean and thus cannot determine how individuals from these regions relate to the ones we analyzed. Third, because we reported few individuals from after 3000 BP, this study provides just a glimpse of the power of this type of analysis to reveal more recent events. Regionally focused studies with large sample sizes are needed to realize the potential of ancient DNA to reveal how the human diversity of this region came to be the way it is today.

## STAR★Methods

### Key Resources Table

**Table:** 

REAGENT or RESOURCE	SOURCE	IDENTIFIER
**Biological Samples**		

Ancient skeletal element	This study	I0308
Ancient skeletal element	This study	I0309
Ancient skeletal element	This study	I2230
Ancient skeletal element	This study	I2232
Ancient skeletal element	This study	I7086
Ancient skeletal element	This study	I7088
Ancient skeletal element	This study	I7090
Ancient skeletal element	This study	I1748
Ancient skeletal element	This study	I8348
Ancient skeletal element	This study	I8349
Ancient skeletal element	This study	I8350
Ancient skeletal element	This study	I3443
Ancient skeletal element	This study	I5456
Ancient skeletal element	This study	I5457
Ancient skeletal element	This study	I9054_d
Ancient skeletal element	This study	I9055_d
Ancient skeletal element	This study	I9056_d
Ancient skeletal element	This study	I9057_d
Ancient skeletal element	This study	I9058_d
Ancient skeletal element	This study	CP18
Ancient skeletal element	This study	CP21
Ancient skeletal element	This study	CP22
Ancient skeletal element	This study	CP23
Ancient skeletal element	This study	CP25
Ancient skeletal element	This study	CP26
Ancient skeletal element	This study	CP19
Ancient skeletal element	This study	LAR001
Ancient skeletal element	This study	LAR002
Ancient skeletal element	This study	MOS001
Ancient skeletal element	This study	I1752
Ancient skeletal element	This study	I1754
Ancient skeletal element	This study	I1753
Ancient skeletal element	This study	I11974
Ancient skeletal element	This study	I2537
Ancient skeletal element	This study	CUN008
Ancient skeletal element	This study	CP8
Ancient skeletal element	This study	CP29
Ancient skeletal element	This study	I2261
Ancient skeletal element	This study	I0237
Ancient skeletal element	This study	I1357
Ancient skeletal element	This study	I1484
Ancient skeletal element	This study	I1485
Ancient skeletal element	This study	I1742
Ancient skeletal element	This study	I2551
Ancient skeletal element	This study	I0039
Ancient skeletal element	This study	I0040
Ancient skeletal element	This study	I0038
Ancient skeletal element	This study	I0041
Ancient skeletal element	This study	I0238

**Chemicals, Peptides, and Recombinant Proteins**		

Pfu Turbo Cx Hotstart DNA Polymerase	Agilent Technologies	Cat# 600412
Herculase II Fusion DNA Polymerase	Agilent Technologies	Cat# 600679
2x HI-RPM hybridization buffer	Agilent Technologies	Cat# 5190-0403
0.5 M EDTA pH 8.0	BioExpress	Cat# E177
Sera-Mag Magnetic Speed-beads Carboxylate-Modified (1 μm, 3EDAC/PA5)	GE LifeScience	Cat# 65152105050250
USER enzyme	New England Biolabs	Cat# M5505
UGI	New England Biolabs	Cat# M0281
Bst DNA Polymerase2.0, large frag.	New England Biolabs	Cat# M0537
PE buffer concentrate	QIAGEN	Cat# 19065
Proteinase K	Sigma Aldrich	Cat# P6556
Guanidine hydrochloride	Sigma Aldrich	Cat# G3272
3M Sodium Acetate (pH 5.2)	Sigma Aldrich	Cat# S7899
Water	Sigma Aldrich	Cat# W4502
Tween-20	Sigma Aldrich	Cat# P9416
Isopropanol	Sigma Aldrich	Cat# 650447
Ethanol	Sigma Aldrich	Cat# E7023
5M NaCl	Sigma Aldrich	Cat# S5150
1M NaOH	Sigma Aldrich	Cat# 71463
20% SDS	Sigma Aldrich	Cat# 5030
PEG-8000	Sigma Aldrich	Cat# 89510
1 M Tris-HCl pH 8.0	Sigma Aldrich	Cat# AM9856
dNTP Mix	Thermo Fisher Scientific	Cat# R1121
ATP	Thermo Fisher Scientific	Cat# R0441
10x Buffer Tango	Thermo Fisher Scientific	Cat# BY5
T4 Polynucleotide Kinase	Thermo Fisher Scientific	Cat# EK0032
T4 DNA Polymerase	Thermo Fisher Scientific	Cat# EP0062
T4 DNA Ligase	Thermo Fisher Scientific	Cat# EL0011
Maxima SYBR Green kit	Thermo Fisher Scientific	Cat# K0251
50x Denhardt’s solution	Thermo Fisher Scientific	Cat# 750018
SSC Buffer (20x)	Thermo Fisher Scientific	Cat# AM9770
GeneAmp 10x PCR Gold Buffer	Thermo Fisher Scientific	Cat# 4379874
Dynabeads MyOne Streptavidin T1	Thermo Fisher Scientific	Cat# 65602
Salmon sperm DNA	Thermo Fisher Scientific	Cat# 15632-011
Human Cot-I DNA	Thermo Fisher Scientific	Cat# 15279011
DyNAmo HS SYBR Green qPCR Kit	Thermo Fisher Scientific	Cat# F410L
Methanol, certified ACS	VWR	Cat# EM-MX0485-3
Acetone, certified ACS	VWR	Cat# BDH1101-4LP
Dichloromethane, certified ACS	VWR	Cat# EMD-DX0835-3
Hydrochloric acid, 6N, 0.5N & 0.01N	VWR	Cat# EMD-HX0603-3

**Critical Commercial Assays**		

High Pure Extender from Viral Nucleic Acid Large Volume Kit	Roche	Cat# 5114403001
MinElute PCR Purification Kit	QIAGEN	Cat# 28006
NextSeq 500/550 High Output Kit v2 (150 cycles)	Illumina	Cat# FC-404-2002
HiSeq 4000 SBS Kit (50/75 cycles)	Illumina	Cat# FC-410-1001/2

**Deposited Data**		

Raw and analyzed data (European nucleotide archive)	This study	ENA: PRJEB28961

**Software and Algorithms**		

Samtools	Li, 2011, Li et al., 2009	http://samtools.sourceforge.net/
BWA	Li and Durbin, 2009	http://bio-bwa.sourceforge.net/
ADMIXTOOLS	Patterson et al., 2012	https://github.com/DReichLab/AdmixTools
SeqPrep	https://github.com/jstjohn/SeqPrep	https://github.com/jstjohn/SeqPrep
bamrmdup	https://bitbucket.org/ustenzel/biohazard	https://bitbucket.org/ustenzel/biohazard
AdapterRemoval v2	Schubert et al., 2016	https://github.com/MikkelSchubert/adapterremoval
Dedup	Peltzer et al., 2016	https://eager.readthedocs.io/en/latest/
smartpca	Patterson et al., 2006	https://www.hsph.harvard.edu/alkes-price/software/
PMDtools	Skoglund et al., 2014	https://github.com/pontussk/PMDtools
Haplofind	Vianello et al., 2013	https://haplofind.unibo.it
Yfitter	https://sourceforge.net/projects/yfitter/	https://sourceforge.net/projects/yfitter/
Schmutzi	Renaud et al., 2015	https://grenaud.github.io/schmutzi/
ANGSD	Korneliussen et al., 2014	https://github.com/ANGSD/angsd
MEGA6	Tamura et al., 2013	https://www.megasoftware.net
mapDamage2.0	Jónsson et al., 2013	https://ginolhac.github.io/mapDamage/

### Contact for Reagent and Resource Sharing

Further information and requests for resources and reagents should be directed to and will be fulfilled by the Lead Contact, David Reich (reich@genetics.med.harvard.edu).

### Experimental Model and Subject Details

#### Archaeological site information

We generated new genome-wide data from skeletal remains of 49 ancient individuals: 15 from Peru, 3 from Belize, 5 from Chile, 11 from Argentina, and 15 from Brazil.

Arroyo Seco 2, Argentina (n = 8)
Laguna Chica, Argentina (n = 3)
Mayahak Cab Pek, Belize (n = 1)
Saki Tzul, Belize (n = 2)
Jabuticabeira 2, Brazil (n = 5)
Lapa do Santo, Brazil (n = 7)
Laranjal, Brazil (n = 2)
Moraes, Brazil (n = 1)
Los Rieles, Central Chile (n = 2)
Conchali, Santiago, Central Chile (n = 2)
Pica Ocho, Northern Chile (n = 1)
Cuncaicha, Highlands, Peru (n = 3)
La Galgada, Highlands, Peru (n = 1)
Laramate, Highlands, Peru (n = 6)
Lauricocha, Highlands, Peru (n = 5)

#### Samples from Argentina

##### Arroyo Seco 2 (AS2): 8960-6950 calBP

• I2230 (SC13_L295_AS2_36): 8960-8380 calBP (AA-24050)
• I2232 (SC7_L204_AS2_49): 8520-8200 calBP (AA-106013)
• I7086 (AS15): 7920-7660 calBP (TO-1503, CAMS-16170, NZA-1101)
• I0309 (ASOB9S14a): 7800-7500 calBP (AA-67738)
• I7088 (AS6): 7570-7290 calBP (LP-186)
• I0308 (ASO_B12_S19): 7570-7300 calBP (AA-9045)
• I1748 (ASOB1S3a) and I7090 (FCS AS2 10497 + FCS AS2 10498): 7330-6950 calBP (AA-7966) [based on one date from the same burial]

The AS2 site is located outside the city of Tres Arroyos, in the Pampas region of Argentina. It is an open-air archaeological site situated on a low-lying knoll between a small temporary lake and a shallow creek (38.36°S, 60.24°W). From 1979 to the most recent excavations in 2015, a total of 77 units (∼314 m^2^) were opened in the site, including shovel tests and 3 long trenches. AS2 is a multicomponent site with several occupation episodes and a chronological range from the Late Pleistocene to historical times (Politis et al., 2016, Pucciarelli et al., 2010).

The earliest evidence for human occupation in the region is ca. 12170 ^14^C years BP (14060 calBP). The hunting/scavenging events of the early hunter-gatherers at the AS2 site likely reflect multiple episodes. Temporary campsites were established in the area for the butchering of now extinct horses (*Equus neogeus* and *Hippidon*) at ca. 11180 ^14^C years BP. During this period, other species of megafauna (*Toxodon*, *Hemiauchenia* and *Glossotherium*) were at the site, although the evidence of human agency is still inconclusive for these taxa. After the extinction of the megamammals there is a gap in the human occupation at Arroyo Seco 2. In Early Holocene times at around 8500 ^14^C years BP, the site was occupied again by guanaco (*Lama guanicoe*) hunters, who established several overlapping camp-sites. Medium and large triangular projectile points, as well a variety of unifacial quartzite tools, characterize the lithic technology during this period. Around this time, funerary activities produced exceptionally abundant human skeletons (n = 50) of both sexes and all age categories, dated between 7819 ± 61 ^14^C years BP and 4487 ± 45 ^14^C years BP (n = 27 dates). The burial modalities are varied, including simple and multiple primary burials and simple and multiple funeral packages. The earliest level of burials included five skeletons with projectile points (midsized triangular, stemless) stuck between and within the bones. Grave goods consisting of marine shell beads and necklaces of canid canines were recorded in some skeletons, indicating an early and complex treatment of the dead.

##### Laguna Chica: 6960-6650 calBP

• I8348 (SC50_L761, LCH.E2-I2.1): 6960-6790 calBP (UCIAMS-185303)
• I8350 (SC50_L763, LCH.E1.3): 6800 calBP [representative date based on other two dates from the site]
• I8349 (SC50_L762, LCH.E2-I1.2): 6780-6650 calBP (UCIAMS-185302)

The Laguna Chica archaeological site is located on the current margins of a small temporary shallow lake in the southeast of the Hinojo-Las Tunas Shallow Lake System. The study area belongs to the Central Pampas Dunefields unit of the aeolian system of central Argentina. Four burials were identified: two in Sector A located in the southern part of the shallow lake and two in Sector B in the west area. The inhumations were dated to the Middle and Late Holocene (Scheifler et al., 2017).

A local farmer discovered Burial N° 1 (36.08°S 62.35°W) in 2006 and then Burial N° 2 in 2008 (both in Sector A). Late in 2016, during systematic archaeological survey, Burials N° 3 and Burial N°4 in Sector B were found. Both burials were partially exposed in the beach of the lake by water erosion. Only the cranium, the mandible and some remains from the thorax region were recovered from Burial N° 1 (sample SC50-L763, LCH.E1.3). The scarce remains of this burial allowed identification of an adult that was morphologically determined to be a probable male (confirmed by our genetic analysis). A decorated pendant made on a canine of a jaguar (yaguareté, *Panthera onca*) was also found associated with this burial.

Burial N° 2 contained two primary burials represented by two individuals who had a dorsal disposition of the bodies with the lower limbs flexed. Individual N° 1 (sample SC50-L762, LCH.E2-I1.2) is an adult female and was dated to 6780-6650 calBP (5930 ± 15 BP, UCIAMS-185302). Individual N° 2 (sample SC50-L761, LCH.E2-I2.1) is an adult male and was dated to 6960-6790 calBP (6080 ± 15 BP, UCIAMS-185303). Although no stratigraphic excavations have been performed in the site, besides the burial, abundant lithic material has been found on the surface along the beach of the lake. These lithic materials are characterized by a predominance of orthoquartzite, followed by other lithic raw materials in low frequencies such as chert, granites, basalt, siliceous chert, and silex, among others. There is a high diversity of tools such as side scrapers, end-scrapers, knives, multipurpose tools, triangular projectile points, and others. In addition, the excavation recovered exhausted orthoquartzite cores. Most lithic raw material came from the Tandilia hill range system (250-350 km to the southeast), but a small quantity of rock came from the Ventania hill range system (170 to the km south), the Tehuelche Mantle (300 km to the southwest), and the Dry Pampas (480 km to the west). The preliminary analysis of the material indicates that the site was occupied during Middle and Late Holocene times. It might represent a succession of residential camps in the border of the pond by hunter-gatherers focused in the exploitation of guanaco (*Lama guanicoe*).

#### Samples from Belize

##### Mayahak Cab Pek and Saki Tzul: 9430-7310 calBP

• I3443 (Burial 6): 9430-9140 calBP (UCIAMS-151854; UCIAMS-151855)
• I5457 (31-P2): 7460-7320 calBP (PSUAMS-3206)
• I5456 (64-P1): 7440-7310 calBP (PSUAMS-3205)

Mayahak Cab Pek (MHCP) and Saki Tzul (ST) are two rockshelter sites located in a remote valley of the Bladen Nature Reserve in the Maya Mountains of southern Belize at approximately 16.49°N, 88.91°W. These sites were excavated in 2014 and 2016 by the Bladen Paleoindian and Archaeological Project (BPAAP) directed by K.M.P., D.J.K., and M.R. The sites both consist of approximately three-meter-deep stratigraphically intact anthropogenic deposits dating from 12500 to 1000 BP.

Burial MHCP14.1.6 consists of an older adult female whose disarticulated remains were interred in a pit approximately 205 cm below the modern ground surface of MHCP. The skeleton was dated on XAD purified amino acids from bulk tissue collagen to 9430-9140 calBP (2σ,UCIAMS 151854 and 151855). Burials ST16.1.2 and ST16.1.3 are both middle-adult males who were interred in flexed positions within the same burial feature, the base of which is located 191 cm below the modern ground surface at ST. ST16.1.2 dated to 7440-7310 calBP (2σ, PSUAMS-3205) and ST16.1.3 dated to 7460-7320 calBP (2σ, PSUAMS-3206). Both ST dates were assays conducted on tooth enamel. FTIR was used to confirm the integrity of the enamel, and several comparative enamel/collagen studies suggest that the age of these ST individuals is likely underestimated by 200 years.

All human remains from Mayahak Cab Pek and Saki Tzul were excavated as part of the Bladen Paleoindian and Archaic Archaeological Project (BPAAP) under permits issued by the Belize Institute of Archaeology (IA) and the Belize Forest Department to carry out archaeological excavations in the Bladen Nature Reserve (BNR), a protected rainforest in Belize. All skeletal remains were exported to the U.S. under permits issued by the IA in accordance with the laws of Belize under the NICH Act and with the explicit permission to conduct molecular analyses on bulk tissues extracted from human bone. BPAAP has a collaborator in Belize, the Ya’axché Conservation Trust (YCT), a local NGO that is the co-manager of the BNR with the Government of Belize. YCT is locally managed and primarily staffed by members of descendent Maya communities. As part of this collaboration, BPAAP research proposals are annually reviewed by the YCT administrative and scientific staff. In 2016 and 2018 K.M.P. gave public consultation presentations to the full staff of YCT and other interested community members regarding ancient DNA, stable isotope, and radiometric dating on tissues extracted from human bones, and in the 2018 consultation the results of the present study were shared. Additionally, in 2017 and 2018 preliminary results of this research were presented at the annual Belize Archaeology Symposium (BAS), a publicly attended venue sponsored by IA. This venue affords the opportunity for both presentation of research results and feedback from the Belizean public.

#### Samples from Brazil

##### Lapa do Santo: 10160-9090 calBP

• CP19 (Lapa01 (Burial 1)): 10160-9600 calBP (Beta-271249)
• CP23 (Lapa22 (Burial 16)): 9550 calBP [representative date based on other five dates from the site]
• CP26 (Lapa05 (Burial 4)): 9550 calBP [representative date based on other five dates from the site]
• CP25 (Lapa15 (Burial 18)): 9680-9530 calBP (MAMS-29425)
• CP22 (Lapa25 (Burial 32)): 9670-9490 calBP (MAMS-17190)
• CP18 (Lapa14 (Burial 15)): 9550-9470 calBP (MAMS-28706)
• CP21 (Lapa24 (Burial 30)): 9410-9090 calBP (MAMS-29423)

Lapa do Santo is an archaeological site located in the northern part of the Lagoa Santa (19.48°S; 44.04°W) karst in east-central Brazil (Strauss et al., 2016, Villagran et al., 2017). Lagoa Santa is well known since the 19th century and is unique in presenting abundant, well-preserved, directly dated, early Holocene human skeletons.

Lapa do Santo is a cave with an associated sheltered area of ca. 1300 m^2^ developed under the overhang of a 30 m high limestone massif. The chronology of the site is based on 21 OSL dates on sediment, 53 radiocarbon dates on charcoal and 20 radiocarbon dates on collagen extracted from human bones. There are three discrete phases of occupation: early Holocene (12700–11700 calBP to 8300–8000 calBP), middle Holocene (5400–4900 calBP to 4300–3900 calBP) and late Holocene (2100–800 calBP to 900–200 calBP) (Strauss et al., 2016).

A total of 39 human burials have been excavated from Lapa do Santo since 2001. Direct dates on bone collagen and stratigraphic observations indicate they all belong to the late phase of the early Holocene component of the site. While the occupation of the site started between 12700–11700 calBP, the use of Lapa do Santo as an interment ground started between 10600-10300 calBP with primary burials. Between 9600-9400 calBP the reduction of the body by means of mutilation, defleshing, tooth removal, exposure to fire and possibly cannibalism, followed by the secondary burial of the remains according to strict rules, became a central element in the treatment of the dead; it seems that these groups were using parts of fresh corpses to elaborate their rituals.

The sediments of the site are mainly anthropogenic, reflecting repeated combustion activities. Multi-proxy analysis indicates a typical early Archaic economy structured around staple carbohydrates complemented by hunting of small and mid-sized animals. The lithic assemblage is dominated by small flakes and cores with crystal quartz as the dominant raw material. While lithic types were constant through time, the use of raw material varied and by ∼9900 calBP non-local sources such as silexite were no longer exploited with the locally available crystal quartz becoming dominant. Low levels of mobility are supported by isotopic and anthropological studies.

##### Laranjal and Moraes: 6900-5660 calBP

• LAR002 (LARAN4 (Burial 3)): 6900-6680 calBP (MAMS-34573)
• LAR001 (LARAN2 (Burial 2)): 6660-6450 calBP (MAMS-34572)
• MOS001 (MORAES2 (Burial 5)): 5910-5660 calBP (MAMS-34575)

Moraes (24.25°S; 47.42°W) and Laranjal (24.28°S; 47.49°W) are two riverine shell middens (i.e., non-coastal) located in the south-eastern region of São Paulo state, in the middle Ribeira Valley. The malacological component of these sites mostly includes the land snail (*Megalobulimus spp*). The material culture includes artifacts made of animal elements (bone, teeth and antler) and shell. Pottery was not produced. The presence of human burials is common in riverine shell middens during the entire Holocene.

The Moraes site (Plens, 2010) is circular with a diameter of approximately 30 m and height of 2 m and is located at the margins of a creek of the same name in the city of Miracatu. The chronology of the site is based on four dates obtained from bone collagen: Burial 3 (KIA-15561, 5895 uncalibrated BP), Burial 37 (KIA-20843, 5420 uncalibrated BP), Burial 5 (KIA-15562, 4985 uncalibrated BP) and Burial 25 (KIA-20844, 4511 uncalibrated BP). In the present study we re-dated Burial 5 to 5092 uncalibrated BP (MAMS-34575).

Laranjal is also located in the city of Miracatu approximately 5 km away from Moraes. The site is circular with a diameter of approximately 20 m and height of 0.6 m and is located on a hilltop 200 m away from a river course, a rare feature for a riverine shell midden (Plens, 2007). The material culture is similar to Moraes. According to a previous radiocarbon date on shell (Beta-189337, 6980 uncalibrated BP), Laranjal represents an early phase of occupation in the region. The new dates on bone presented here point to a slightly younger age for the site: Burial 2 (6585 uncalibrated BP) and Burial 3 (6849 uncalibrated BP).

##### Jabuticabeira 2: 2360-1080 calBP

• I9058_d (MPI 25 (Burial 10B)): 2360-2160 calBP (MAMS-28361)
• I9057_d (MPI 24 (Burial 110)): 2340-2100 calBP (MAMS-28358)
• I9054_d (MPI 21 (Burial 111/112)): 2030-1830 calBP (MAMS-28365)
• I9055_d (MPI 22 (Burial 111/112)): 1990-1750 calBP (MAMS-28365)
• I9056_d (MPI 23 (Burial 102)): 1290-1080 calBP (MAMS-28359)

Sedentary people constructed many hundreds of shell mounds between circa 8000 and 1000 BP on the Brazilian coast, a practice known as the Sambaqui tradition. The mounds consisted of inorganic sediment, mollusk shells, food debris and organic matter (including burials), fish offerings, and stone artifacts. The sites varied in size and some of them had monumental aspects, reflecting hierarchies among regional settlements and rising social complexity (DeBlasis et al., 2007).

Dental samples of five individuals from a shell mound (28.55°S; 49.01°W) were included in this study. They were selected from the more than 200 excavated inhumations at the monumental Sambaqui Jabuticabeira 2 site in southeastern Brazil dated to 3137–2794 to 1860–1524 calBP - 2σ (DeBlasis et al., 2007). Craniometric studies at this and many other shell mounds in Brazil reveal a morphological pattern different from that of earlier groups that inhabited the inland, consistent with genetic evolution or alternatively movements and mixtures of coastal populations (Hubbe et al., 2009).

#### Samples from Chile

##### Los Rieles: 11140-4870 calBP

• I11974 (LV036 (individual 1)): 11140-10730 calBP (UCIAMS-79662)
• I1753 (LV036 (individual 2)): 5310-4870 calBP (UGAMS-04600, Beta-254447)

The Los Rieles site (31.92°S; 71.50°W; 20 m above sea level) from North-Central Chile is an extensive stratified shell-midden with material culture evidence ranging from 12400 calBP to 4850 calBP and burials of six individuals (Jackson et al., 2012).

One sample (#1) analyzed in this study is derived from an incomplete male adult (40-45 years old) buried in a semi-flexed lateral position from a stratigraphic unit underlaying the shell midden. This individual has been directly dated to at least 11140-10730 cal BP (UCIAMS-79662) after applying a local marine reservoir correction to the most recent radiocarbon date obtained from the petrous bone, the same anatomical element from which DNA was extracted. Other available dates on teeth for this individual are roughly the same age, although the radiocarbon fraction measurements are statistically inconsistent at α = 0.05, possibly reflecting differences in the chemical pretreatments at different laboratories prior to dating (uncalibrated dates of 9815 ± 30 BP (UCIAMS-79662) for the petrous bone, 10150 ± 30 BP (UGAMS-4599) for a tooth; and 10470 ± 60 BP (BETA-251901) for another tooth) (Ward and Wilson, 1978). Stable isotope values of δ^13^C = −13.5‰ and δ^15^N = 18.0‰ were obtained in association with one of the dates (UGAMS-04599). This individual has yielded the earliest directly-dated human bone material from South America (Jackson et al., 2012).

A second individual (#2) is an almost complete male adult (24-26 years old) excavated from a burial pit below the shell midden stratum. It is directly dated to 5310-4870 calBP based on two statistically indistinguishable dates (UGAMS-04600, Beta-254447). Stable isotope values of δ^13^C = −13.2‰ and δ^15^N = 16.2‰ were obtained in association with one of them (Beta-254447).

Stable isotope results for both individuals are consistent with broad-spectrum diets, heavily relying on marine protein (Jackson et al., 2012).

##### Conchalí: 910-540 calBP

• I1754 (ConchaliIND2): 910-740 calBP (Poz-83481)
• I1752 (ConchaliIND1): 650-540 calBP (UGAMS-3241)

The Conchalí site (33.37°S; 70.67°W; 510 m above sea level), within the city of Santiago, was identified during urban construction when the remains of two individuals (#1: ∼600 calBP; #2 ∼830 calBP) were excavated. Both individuals are males and their bones show overall good preservation, although there was no direct association to any other archaeological material.

##### Pica Ocho: 720-570 calBP

• I2537 (PO2): 720-570 calBP (PSUAMS-1870)

Pica-8 is a Late Intermediate Period (AD 900–1450) cemetery located in the Pica-Matilla oasis in the Atacama Desert at 1,350 m of altitude (20.51°S, 69.33°W). The complex belongs to the Late Intermediate Period (LIP). Even though Pica 8 is located approximately 90 km from the coast, the archaeological evidence from this cemetery, as with other similar sites, suggests connections between the oases and the seaside.

#### Samples from Peru

##### Cuncaicha, Peru: 9240-3180 calBP

• CP29 (Cuncaicha 15-07): 9240-8770 calBP (AA105087, AA-107847)
• CP8 (Cuncaicha 15-03): 4290-4080 calBP (AA-107842, AA-109149)
• CUN008 (Cuncaicha 15-04): 3370-3180 calBP (AA109414, AA109416)

Cuncaicha rockshelter (15.39°S 72.61°W, 4480 m above sea level) is the highest well-dated Terminal Pleistocene site in the Andes (Rademaker et al., 2014). Archaeological investigations have documented episodic residential occupation by hunter-gatherers beginning ∼12500-12000 calBP. By ∼9000 years ago, the site also became a cemetery where hunter-gatherers, and later pastoralists, interred their dead. Episodes of occupation alternated with episodes of burial until the Late Holocene (M.F., J.B., H.R.-C., K.H., and K.R., unpublished data; K.R. and G. Hodgins, unpublished data). Stable isotope analyses of five burials spanning the Early to Late Holocene, including those yielding the genomes discussed in this study, indicate that these people were permanent highlanders (Chala-Aldana et al., 2018). Due to the cold mean annual temperature at this elevation and dry, protected conditions in the rock shelter sediments, these and other skeletons from Cuncaicha generally exhibit excellent preservation. This paper includes the first ancient DNA data from Cuncaicha, from one Early Holocene and two Late Holocene individuals. Cuncaicha 15-07, a gracile female 18 to 25 years old, is a nearly complete extended burial. Two ultrafiltered (UF) collagen AMS ages obtained on a radius and tibia average to a 95% range of 9240-8770 calBP. Cuncaicha 15-03, a robust male 30-50 years of age, is a seated, flexed burial. Two UF AMS ages on a fibula and tooth give a date of 4290-4080 calBP and genomic analyses confirmed that those two anatomical elements belong to the same individual (data not shown). Cuncaicha 15-04, a robust male 30-55 years of age, is a partial, flexed burial. Two UF AMS ages on the left and right fibulae average 3370-3180 calBP.

##### Cueva Lauricocha: 8730-3450 calBP

• I0038 (LAU2): 8730-8520 calBP (MAMS-14391)
• I0238 (LAU1): 8560 calBP [representative date based on other two dates from the site]
• I0041 (LAU5): 8580-8420 calBP (MAMS-14731)
• I0040 (LAU4): 5940-5740 calBP (MAMS-14390)
• I0039 (LAU3): 3610-3450 calBP (MAMS-14389)

Lauricocha is a rock shelter in the Huanuco Province of Peru, located at ∼4050 m above sea level near Lake Lauricocha and the source of the Marañón River (10.32°S 76.67°W). The site was excavated by Augusto Cardich in several campaigns between 1958 and the early 1960’s and revealed incomplete skeletal remains of 11 humans (8 adults, 3 sub-adults) along with stone tools and burnt and unburnt animal bones in the lower layers of the site stratigraphy (Cardich, 1964). Based on contextual radiocarbon dates the burials were first dated to about ∼10000 calBP, however reinvestigations of the skeletal remains revealed that the burials are not all contemporary and that burial dates range from the Early Archaic (∼8600 calBP) to the Initial Period (∼3500 calBP). For more details see Fehren-Schmitz et al. (2015).

##### La Galgada: 4230-3980 calBP

• I2261 (SC10_L259_LGA30): 4230-3980 calBP (MAMS-27354)

La Galgada was a ceremonial and administrative site in the Northern Peruvian highlands dating to the Late Archaic and Initial Period (∼4700-3500 calBP) and associated with the Kotosh Religious tradition (8.47°S 78.15°W). While situated in the highlands of the Department of Ancash, the site is found at a relatively low altitude of ∼1100 m above sea level, built in the Tablachaca canyon at the shores of the river of the same name. The site is dominated by two mounts with monumental stone architecture, including stone chambers of which some were also used for burials. For more details see Grieder et al. (1988). The sample for which we report genomic data here derives from an older male found as one of four individuals in the Late Archaic chamber D11-C3.

##### Yacotogia (PAP-854), Laramate, Highlands: 1160-960 calBP

• I1485 (YA5): 1160-960 calBP (MAMS-12302)

The site is located on the left bank of the Llauta River, in a high area of Cerro Sausana at almost 3400 m above sea level, where there is a series of rocky outcrops that make up several caves and natural shelters. Two of these have been especially intensively used for funerary purposes during the Middle Horizon and Late Intermediate Period (14.26°S, 74.86°W).

The two funerary shelters are located at the base of volcanic rock cliffs, on both sides of a ravine. The largest shelter occupies the east side of the ravine (it measures 9.20 m long at the entrance x 10 m deep and 85 cm high) and inside were the skeletal remains of more than 60 individuals whose skeletons were disturbed after death. The smallest shelter occupies the western side of the ravine, has a triangular shape and measures 3.70 m long at the entrance, 2.80 m high and 7.50 m deep. Inside were only a few human remains.

##### Botigiriayoq (PAP-784), Laramate, Highlands: 920-730 calBP

• I0237 (BC12): 920-800 calBP (PSUAMS-1614)
• I1357 (BC14): 880 calBP [representative date based on other four dates in the Laramate region]
• I1484 (BC13): 900-730 calBP (PSUAMS-1615)

Botigiriayoq is located in the high part of an elongated plateau that forms the dividing line between the rivers Llauta (Palpa) and Laramate (Viscas), and is situated at around 3500 m above sea level (14.28°S, 74.84°W). It is a fairly large site that is divided into three sectors (A, B and C), with a scattered occupation that includes the slopes of three hills located at the southern end of the plateau. Sector B, from which the analyzed samples come, is located in the middle of the three hills, which are made up of Cretaceous material. The site contains numerous rock shelters of different dimensions that were used as funerary shelters. These are grouped or dispersed in the high parts of the hills and several have been reused in more recent times by local pastoralists. On the north side of the site, there are another 5-6 rock shelters that were used for multiple burials of children and adults, all of which have been looted. The Late Intermediate period is the most probable occupation time.

##### Huayuncalla (PAP-767), Laramate, Highlands: 910-740 calBP

• I2551 (HA1): 910-740 calBP (PSUAMS-1603)

The site of Huayuncalla is located on the left bank of the Laramate river, around 3100 m above sea level. It is on an elongated hill that projects almost from northeast to southeast, descending from the highest hills toward the Laramate river (14.26°S, 74.86°W). The site occupies more than six hectares with distinct sectors of stone buildings, including oval and circular enclosures, as well as large quadrangular enclosures, room terraces, funerary structures, and large patios or spaces delimited by alignments of large stones. The site was occupied during the Late Paracas period, the Middle Nasca period and the Wari era.

The largest concentration of structures is observed in the highest part of the site, where there is a large group of oval and circular enclosures arranged at the top and on two levels of terraces to the south side. In the flattest part of the summit there are remnants of straight walls that delimit rectangular enclosures, which alternate with courtyards or spaces free of buildings. At the highest point, there are two enclosures with circular floors surrounded by an enclosure with a rectangular floor plan; these were important funerary enclosures. Rectangular plan constructions are also found on the northwest side of the site. Excavations have revealed rectangular-shaped enclosures from the Wari era.

In a lower level of the north side are three funerary structures (known as kuntis), two rectangular and one irregular oval, located next to each other. One of the two rectangular structures is larger and has three interior compartments. All the funerary structures were looted and have collapsed with the passage of time.

##### Tranca (PAP-879), Laramate, Highlands: ∼880 calBP

• I1742 (TA12): 880 calBP [representative date based on other four dates from the site]

The site of Tranca is located on the left bank of the Laramate River at 2800 m above sea level, and occupies a site that slopes gently from Cutamalla Hill to the Laramate River in the Santa María sector (14.26°S, 74.86°W). It is a residential type site that includes isolated tombs and agricultural terraces, occupied during the Middle Horizon (∼1300-950 calBP).

The site comprises four to five groups of dispersed housing terraces in an area of more than two hectares. In the upper part of the site there is a group of terraces where the foundations of six small circular structures can be observed. Further to the northeast is a 6 × 7 m rectangular enclosure with high walls, small niches and remains of lintels, as well as other poorly preserved terraces. The walls are built with a double row of stones joined with clay mortar that preserve regular walls. At the western end are the remains of a D-shaped structure built with blocks of selected rectangular stone arranged in two rows with internal land fill. Finally, in the lower part of the site there are remains of four possible tombs.

At the east end there is an oval-shaped funerary structure 3.5 × 5.2 m on each side, divided into two equal spikes and connected by a 1.0 m wide and 50 cm high access. The walls are built with stones of different sizes and have a ceiling in the form of a false vault. Inside the tomb were remains from more than 30 individuals. Finally, in the lower part, at the west end of the site and in the last two levels of terraces, are the remains of four small looted tombs of 1.5 m in diameter.

#### Grouping ancient samples into analysis clusters

For most analyses, we grouped samples by site and time period, leading to the “Main analysis label” specified in a column of Table S3. For several analyses we also considered larger pools of samples as indicated by the “Regional Label” specified in Table S3. In particular, we pooled northern Chilean and southern Peruvian samples after ∼4200 BP into a “*Late Central Andes*” category based on their distinctive pattern of allele sharing with ancient North Americans compared to other ancient South Americans. As a contrast, we also defined an “*Early Andes*” category that included Central Andes samples earlier than ∼4200 BP as well as all the North Andes samples in our dataset which includes samples that date to the millenium following ∼4200 BP (*Peru_Lauricocha_3500BP* and *Peru_LaGalgada_4100BP*) because of the evidence of local continuity in these samples based on *f*-statistic and *qpWave* analysis.

### Method Details

#### Direct AMS ^14^C bone dates

We report 31 new direct AMS ^14^C bone dates from eleven radiocarbon laboratories (Arizona [AA] – 1; Mannheim [MAMS] – 18; Poznan [Poz] – 1; Pennsylvania State University [PSUAMS] – 6; UC Irvine [UCIAMS] - 4; University of Georgia [UGAMS] – 1) and recalibrate 23 previously published radiocarbon dates (Table S3). Bone preparation and quality control methods for most of these samples are described elsewhere and the details can be found on laboratory-specific websites. Detailed methods are provided below for PSUAMS, UCIAMS and MAMS.

#### PSUAMS and UCIAMS Protocols and Pretreatment

##### Ultrafiltration

At PSUAMS and UCIAMS, bone collagen for ^14^C and stable isotope analyses was extracted and purified using a modified Longin method with ultrafiltration (Kennett et al., 2017). Bones were initially cleaned of adhering sediment and the exposed surfaces were removed with an X-acto blade. Samples (200–400 mg) were demineralized for 24–36 h in 0.5N HCl at 5°C followed by a brief (< 1 h) alkali bath in 0.1N NaOH at room temperature to remove humates. The residue was rinsed to neutrality in multiple changes of Nanopure H_2_O, and then gelatinized for 12 h at 60°C in 0.01N HCl. The resulting gelatin was lyophilized and weighed to determine percent yield as a first evaluation of the degree of bone collagen preservation. Rehydrated gelatin solution was pipetted into pre-cleaned Centriprep ultrafilters (retaining 430 kDa molecular weight gelatin) and centrifuged 3 times for 20 min, diluted with Nanopure H_2_O, and centrifuged 3 more times for 20 min to desalt the solution.

##### XAD Amino Acids

In some instances, collagen samples were too poorly preserved and were pre-treated at Penn State using a modified XAD process (Lohse et al., 2014). Samples were physically cleaned using hand tools and sectioned with disposable Dremel cut-off wheels and then demineralized in 0.5 N HCl for 2-3 days at 5°C. The demineralized collagen pseudomorph was gelatinized at 60°C in 1-2 mL 0.01 N HCl for eight to ten hours. Sample gelatin was pipetted into a pre-cleaned 10 mL disposable syringe with an attached 0.45 mm Millex Durapore PVDF filter (precleaned with methanol and Nanopure H2O) and driven into a thick-walled culture tube. The filtered solution was lyophilized and percent gelatinization and yield determined by weight. The sample gelatin was then hydrolyzed in 2 mL 6 N HCl for 22 h at 110°C. Supelco ENVI-Chrom® SPE (Solid Phase Extraction; Sigma-Aldrich) columns were prepped with 2 washes of HCl (2 mL) and rinsed with 10 mL DI H2O. With a 0.45 mm Millex Durapore filter attached, the SPE Column was equilibrated with 50 mL 6 N HCl and the washings discarded. 2 mL collagen hydrolyzate as HCl was pipetted onto the SPE column and driven with an additional 10 mL 6 N HCl dropwise with the syringe into a 20 mm culture tube. The hydrolyzate was finally dried into a viscous syrup by passing UHP N_2_ gas over the sample heated at 50°C for ∼12 h.

#### PSUAMS and UCIAMS Quality Control and Measurement

Carbon and nitrogen concentrations and stable isotope ratios of the XAD amino acid samples were measured at the Yale Analytical and Stable Isotope Center with a Costech elemental analyzer (ECS 4010) and Thermo DeltaPlus analyzer (Kennett et al., 2017). Sample quality was evaluated by % crude gelatin yield, %C, %N and C/N ratios before AMS ^14^C dating. C/N ratios for all samples fell between 2.9 and 3.6, indicating good collagen preservation. Samples (∼2.1 mg) were then combusted for 3 h at 900°C in vacuum-sealed quartz tubes with CuO and Ag wires. Sample CO_2_ was reduced to graphite at 550°C using H_2_ and a Fe catalyst, with reaction water drawn off with Mg(ClO_4_)_2_. Graphite samples were pressed into targets in Al boats and loaded on a target wheel with OX-1 (oxalic acid) standards, known-age bone secondaries, and a ^14^C-free Pleistocene whale blank. ^14^C measurements were at UCIAMS on a modified National Electronics Corporation compact spectrometer with a 0.5 MV accelerator (NEC 1.5SDH-1). The ^14^C ages were corrected for mass-dependent fractionation with measured δ^13^C values and calibrated with samples of Pleistocene whale bone (backgrounds, 48000 ^14^C BP), late Holocene bison bone (∼1,850 ^14^C BP), late AD 1800s cow bone and OX-2 oxalic acid standards.

#### PSUAMS Acid Etch/Hydrolysis

Enamel bioapatite splits from two samples from Saki Tzul, Belize (I5456 and I5457) were processed at the Pennsylvania State University for AMS ^14^C dating by acid hydrolysis. Samples were cleaned with dental tools to remove adhering residues, and then acid-etched to removed secondary carbonate prior to hydrolysis. After rinsing in Nanopure H_2_O and drying at 50°C, samples were evaluated for the integrity of their enamel using FTIR analysis. Samples and standards were placed then in BD Vacutainer septum-stopper vials, and digested with 85% orthophosphoric acid. The evolved CO_2_ was graphitized as above and the ^14^C measurement made on a modified National Electronics Corporation compact spectrometer with a 0.5 MV accelerator (NEC 1.5SDH-1) at Penn State University.

#### MAMS Protocols and Pretreatment

The samples from Lapa do Santo MAMS-28703, −28706, and −17190 were pretreated at the Department of Human Evolution at the Max Planck Institute for Evolutionary Anthropology (MPI-EVA), Leipzig, Germany, using the method described in Talamo and Richards (2011). The outer surface of the bone samples was first cleaned by a shot blaster and then 500 mg of the whole bone was taken. The samples were then decalcified in 0.5M HCl at room temperature until no CO_2_ effervescence was observed, usually for about 4 hours. 0.1M NaOH was added for 30 minutes to remove humic acids. The NaOH step was followed by a final 0.5M HCl step for 15 minutes. The resulting solid was gelatinized at pH3 in a heater block at 75°C for 20h. The gelatine was then filtered in an Eeze-Filter (Elkay Laboratory Products (UK) Ltd.) to remove small (> 80 μm) particles. The gelatine was then ultrafiltered with Sartorius “VivaspinTurbo” 30 KDa ultrafilters. Prior to use, the filter was used to remove carbon containing humectants. The samples were lyophilized for 48 hours. All dates were corrected for a residual preparation background estimated from ^14^C free bone samples. These bones were kindly provided by the Mannheim laboratory and pretreated in the same way as the archaeological samples (Korlević et al., 2018). To assess the preservation of the collagen, C:N ratios together with isotopic values need to be evaluated. The C:N ratio should be between 2.9 and 3.6 and the collagen yield not less than 1% of the weight. Stable isotopic analysis is evaluated at MPI-EVA, Leipzig (Lab Code R-EVA), using a ThermoFinnigan Flash EA coupled to a Delta V isotope ratio mass spectrometer. All the samples fall within the acceptable range of the evaluation criteria mentioned above.

#### Calibration of radiocarbon dates

All calibrated ^14^C ages were calculated using OxCal version 4.3 (Ramsey and Lee, 2013). The IntCal13 northern hemisphere curve (Reimer et al., 2013) was used for four samples from Belize, while the remainder were calibrated using the SHCal13 curve (Hogg, 2013). Dates from two coastal sites—Los Rieles in Chile and Jabuticabeira II in Brazil—were calibrated according to a mixture with the Marine13 curve (Reimer et al., 2013) based on an estimate of a 40% marine dietary component. For each site, ΔR values were calculated based on the most proximate sample locations in the 14CHRONO Marine Reservoir Database (Reimer and Reimer, 2001) (see Table S3 for details).

To define genetic group labels we in general used the following nomenclature: “*Country_SiteName_AgeBP*” (Eisenmann et al., 2018). “*AgeBP*” of a genetic group comprised of more than one individual is calculated by averaging the mean calibrated date in years before present (BP) of the directly dated samples that provided nuclear DNA data. For samples that were not directly dated we considered the averaged value of the corresponding genetic group.

#### Ancient DNA sample processing

We screened skeletal samples for DNA preservation in dedicated clean rooms at Harvard Medical School in Boston (USA), UCSC Paleogenomics in Santa Cruz (USA), the Max Plank Institute for Science of Human History in Jena (Germany), the University of Tübingen in Tübingen (Germany) and the Australian Centre for Ancient DNA in Adelaide (Australia) (Table S3). Powder was prepared from the skeletal samples and DNA extraction (Dabney et al., 2013) and library preparation (Rohland et al., 2015) were performed using previously established protocols. Except for samples processed with the single-stranded library protocol (*Brazil_Jabuticabeira2_2000BP*) (Gansauge and Meyer, 2013), all other samples were treated with uracil-DNA glycosylase (UDG) to greatly reduce the presence of errors characteristic of ancient DNA at all sites except for the terminal nucleotides (Rohland et al., 2015), or including at the terminal nucleotides (UDGplus) (Briggs et al., 2010). We enriched for sequences overlapping 1,233,013 SNPs (‘1240k SNP capture’) (Fu et al., 2015) and sequenced the DNA on Illumina NextSeq500 or HiSeq 4000 instruments. We removed adapters from the sequences using *SeqPrep* (https://github.com/jstjohn/SepPrep) or *AdapterRemoval v2* (Schubert et al., 2016), mapped the data to hg19 with BWA (Li and Durbin, 2009), removed duplicates with bamrmdup (Stenzel, 2018) or Dedup (Peltzer et al., 2016) and merged data from different libraries of the same individual using *SeqPrep*. The damage patterns were quantified using mapDamage2.0 (Jónsson et al., 2013). We extracted genotypes from the ancient genomes by drawing a random sequence at each position, ignoring the first and last 2 bp of every read as well as any read containing insertions or deletions in their alignment to the human reference genome. For samples not treated with UDG (UDGminus) (e.g., *USA_Anzick1_11400BP* and *Brazil_Jabuticabeira2_2000BP*), we also clipped 10bp and created a second dataset to represent the sample following this processing. If the randomly drawn haploid genotype of an ancient individual did not match either of the alleles of the biallelic SNP in the reference panel, we set the genotype of the ancient individual as missing. For the great majority of analyses we analyzed all autosomal SNPs from the ∼1.2 million SNP enrichment reagents. For a subset of analyses we restricted to transversion SNPs which are unaffected by the characteristic ancient DNA errors that occur at transition SNPs.

### Quantification and Statistical Analysis

#### Contamination estimation in mitochondrial DNA, the X chromosome, and the autosomes

We tested for contamination in mtDNA using *schmutzi* (parameters:–notusepredC–uselength) (Renaud et al., 2015), which iteratively determines the endogenous mitochondrial genome while also estimating human mitochondrial contamination given a database of potential contaminant mitochondrial genomes. For males we estimated contamination on the X chromosome with ANGSD (Korneliussen et al., 2014), which creates an estimate based on the rate of heterozygosity observed on the X chromosome. We used the parameters minimum base quality = 20, minimum mapping quality = 30, bases to clip for damage = 2, and set all other parameters to the default. Finally, we measured autosomal contamination using a recently developed tool based on breakdown of linkage disequilibrium that works for both males and females (N.N., Éadaoin Harney, S.M., N.P., and D.R., unpublished data). We report but do not include in our main analyses samples with evidence of contamination greater than 5% by any of the contamination estimation methods (only sample CP26 was excluded). Due to high contamination levels (the non-damage restricted samples skewed toward West Eurasians on global PCA (not shown), sequences of all *Brazil_Jabuticabeira2_2000BP* samples were filtered with PMDtools (Skoglund et al., 2014) to retain only fragments with a typical ancient DNA signature and then trimmed 10bp on either end before analysis. All contamination estimates are reported in Table S3.

#### Present-day human data

We used present-day human data from the Simons Genome Diversity Project (Mallick et al., 2016), which included 26 Native American individuals from 13 groups with high coverage full genome sequencing. We also included data from 48 Native American individuals from 9 different populations genotyped on the Affymetrix Human Origins array (Lazaridis et al., 2014, Skoglund et al., 2015) as well as 493 Native American individuals genotyped on Illumina arrays either unmasked or masked to remove segments of possible European and African ancestry (Reich et al., 2012).

#### Y chromosome and mitochondrial DNA analyses

For Y chromosome haplogroup calling, we used the original BAM files and performed an independent processing procedure. We filtered reads with mapping quality < 30 and bases with base quality < 30, and for UDGhalf treated libraries we trimmed the first and last 2-3bp of each sequence to remove potential damage induced mutations. We determined the most derived mutation for each sample using the tree of the International Society of Genetic Genealogy (ISOGG) and confirmed the presence of upstream mutations consistent with the assigned Y chromosome haplogroup using Yfitter (Jostins et al., 2014). For mtDNA haplogroup assignment, we used *Haplofind* (Vianello et al., 2013) on the consensus sequences reconstructed with *schmutzi* (parameters:–notusepredC–uselength) (Renaud et al., 2015) after applying a quality filter of ≥ 10 (or ≥ 11 for LapaDoSanto_Burial28, LapaDoSanto_Burial17 and ArroyoSeco2_AS6) for a total of 48 newly reported sequences, including samples for which no nuclear data was obtained (Table S3). We produced a multiple genome alignment of our newly reconstructed sequences (excluding *LagunaChica_SC50_L763* because of low coverage) along with 17 previously published ancient sequences older than ∼4000 BP (Table S3) and 230 present-day sequences (Llamas et al., 2016) using MUSCLE (parameter: -maxiters 2) (Edgar, 2004). We thus analyzed a total of 295 mtDNAs and used an African sequence as outgroup. We used the program MEGA6 (Tamura et al., 2013) to build a Maximum Parsimony tree with 98% partial deletion (16518 positions) and 500 bootstrap iterations, and visualized it in FigTree (http://tree.bio.ed.ac.uk/software/) (Figure S7).

We used the newly reconstructed mtDNA combined with previously published present-day and ancient sequences (Table S3) to generate a maximum parsimony tree (Figures S7A and S7B). This tree recapitulates the star-like phylogeny of the founding Southern Native American mtDNA haplogroups A2, B2, C1b, C1c, C1d, D1 and D4h3a reported previously (Tamm et al., 2007). We report five new Central and South American individuals belonging to the rare haplogroup D4h3a (3 Brazil, 1 Chile, 1 Belize), which among ancient individuals has been identified so far only in two individuals from the North American Northwest Coast (Lindo et al., 2017) and in the *Anzick-1* individual (Rasmussen et al., 2014) but not in Southern Ontario, ancient Californians (Scheib et al., 2018), or Western South America (Llamas et al., 2016) where it has the highest frequency today (Perego et al., 2009). Previously this haplogroup was hypothesized to be a possible marker of human dispersal along the Pacific coast, but its presence in early individuals from Belize and Brazil (as well as in the inland *Anzick-1* genome from Montana in the U.S.A.) suggests an ancient spread toward the Atlantic coast as well with its lower frequency there today being due to population replacement or to genetic drift.

The maximum parsimony tree is also striking in showing that the lineage leading to haplogroup D4h3a has a much longer branch than all other Native American-specific mtDNA haplogroups. The diversification of haplogroup D4h3a dates to ∼16000 BP which temporally overlaps with the coalescence time of A2, B2, C1, and D1 haplogroups (Llamas et al., 2016). This suggests that a rate acceleration took place on the lineage leading to the radiation of D4h3a, similar to what has been observed among African L2 lineages (Torroni et al., 2001).

#### Principal component analysis

We used *smartpca* from EIGENSOFT and default settings (Patterson et al., 2006) to compute principal components using present-day populations. We projected ancient individuals with at least ∼10,000 overlapping SNPs using the option *lsqproject:* YES, on eigenvectors computed using the present-day populations genotyped on the Illumina array (we restricted our analysis to the subset of Native Americans without evidence of post-colonial mixture (Reich et al., 2012)).

#### Symmetry statistics and admixture tests (f-statistics)

We computed *D*-statistics, *f*_*4*_-statistics and *f*_*3*_-statistics with ADMIXTOOLS (Patterson et al., 2012) using the programs *qp3Pop* and *qpDstat* with default parameters and “f4mode: YES.” We computed standard errors with a weighted block jackknife over 5-Mb blocks. For *f*_*3*_-statistics we set the “inbreed: YES” parameter to account for the fact that we are representing the ancient samples by a randomly chosen allele at each position rather than using their full diploid genotype which we do not have enough data to discern. The details of the inbreeding correction, which computes the expected value of statistics taking into account this random sampling, are presented in the section 1.1 of the Appendix of Reich et al. (2009). We computed “outgroup” *f*_*3*_-statistics of the form *f*_*3*_*(Mbuti; Pop*_*1*_*, Pop*_*2*_*),* which measures the shared genetic drift between population 1 and population 2. Where relevant we plot the statistics on a heatmap using R (https://github.com/pontussk/point_heatmap/blob/master/heatmap_Pontus_colors.R). We also created a matrix of the outgroup f_3_ values between all pairs of populations. We converted these values to proxies for distances by subtracting the values from 1 and generating a multi dimensional scaling (MDS) plot with a custom-made R script. We converted the original values to distances by taking the inverse of the values and generating a Neighbor joining tree using PHYLIP version 3.696’s (Felsenstein, 1989) “neighbor” function and setting *USA_USR1_11400BP* as the outgroup (default settings were used for the rest of the analysis). We displayed the tree using Itol (Letunic and Bork, 2011).

#### *qpWave* analyses

To determine the minimum number of streams of ancestry contributing to Central and South American populations, we used the software *qpWave* (Reich et al., 2012) which assesses whether the set of *f*_*4*_-statistics of the form *f*_*4*_*(A = South American 1, B = South American 2; X = outgroup 1, Y = outgroup 2)*, which is proportional to the product of allele frequencies summed over all SNPs *(p*_*A*_*-p*_*B*_*)(p*_*X*_*-p*_*Y*_*)*, forms a matrix that is consistent with different ranks (rank 0 would mean consistency with a single stream of ancestry relative to the outgroups; rank 1 would mean 2 streams of ancestry, and so on). The significance of the statistic is assessed using a Hotelling T^2^ test that appropriately corrects for the correlation structure of *f*_*4*_-statistics (and thus multiple hypothesis testing). For most analyses, we used ancient California individuals from Scheib et al. (2018) (*USA_MainlandChumash_1400BP, USA_SanFranciscoBay_300BP, USA_SanNicolas_4900BP, and USA_SanClemente-SantaCatalina_800BP*), *Chipewyan, Russia_MA1_24000BP (MA1), Anzick-1, Han, Papuan, Karelia Hunter Gatherer*, and modern Mexican groups (*Zapotec, Mixtec, Mixe,* and *Mayan*) as outgroups. We also performed the analyses with different outgroups to determine the effect of outgroups on the results (for a detailed list, see Table S5). We used all possible pairs, triplets, and quadruplets of South American groups as test populations. We also tried different combinations of South American groups—up to 15 different groups together—as test populations. For *qpWave* analyses we used the default settings except for the change that we set allsnps: YES.

#### Admixture graph modeling

We used *qpGraph* (Patterson et al., 2012) to model the relationships between diverse samples. This software assesses the fit of admixture graph models to allele frequency correlation patterns as measured by *f*_*2*_, *f*_*3*_, and *f*_*4*_-statistics. We started with a skeleton phylogenetic tree consisting of *Mbuti, Russia_MA1_24000BP (MA1*), *Onge*, and *Han* from prior publications (Lipson and Reich, 2017, Skoglund et al., 2015). We added the ancient South American populations in different combinations and retained only the graph solutions that provided no individual *f*_*4*_-statistics with |Z| > 3.5 between empirical and predicted statistics (except for the case of adding *Surui* due to the difficulties of modeling in the *Population Y* signal). We created the graphs with all overlapping SNPs among the included groups. We used the default settings of *qpGraph* for all runs exept for the options “outpop: NULL” instead of setting an outgroup population and “allsnps: YES” to compute each *f*-statistic on the common SNPs present in the populations involved in the statistic, rather than the intersection of all SNPs present in the dataset. To reduce the impact of damage-induced substitutions in UDGminus data of the *Anzick-1* individual we restricted the analysis to a version of this sample where sequences were 10bp trimmed on both sides before genotyping. In addition, we performed all analyses with the transitions at CpG sites removed, and we also report the maximum Z-scores of many of the analyses with all transition sites removed. Lastly, for the graphs in Figures S6A-D we computed standard errors for the lengths of different graph edges by performing a block jackknife by dropping each of 100 contiguous blocks (with an equal number of SNPs) in turn (Lazaridis et al., 2016).

Scheib et al. analyzed data from diverse Native American populations—ancient and modern—and proposed that in Central and South Americans today there is a history of widespread admixture between the two deepest branches of Native American genetic variation (*ANC-A* and *ANC-B*), with a minimum of ∼30% of each branch admixed into all populations (Scheib et al., 2018). They write “The summary of evidence presented here allows us to reject models of a panmictic “first wave” population from which the ASO [the Ancient South Ontario population] diverged after the population of South America or in which solely the ANC-A population contributed to modern southern branch populations.”

The evidence for the claim that Central and South Americans do not have entirely *ANC-A* ancestry is based on fitting the admixture graph model of Figure 2A in Scheib et al. (2018), which the authors show is a fit to the data jointly for *Han, Anzick-1, USA_SanNicolas_4900BP (ESN), USA_SanNicolas_1400BP (LSN), Pima, Surui,* and *Canada_Lucier_4800BP-500BP (ASO)*. They then added a diverse set of other Native American populations into the graph as mixtures of the same two lineages, and report the mixture proportions in Table S8 of their study.

We began by replicating the finding of Scheib et al. (2018) that their proposed admixture graph was a fit to the data (maximum mismatch between observed and expected *f-*statistics of |Z| = 1.1) (Figure S6A). However, when we added to the admixture graph additional non-American populations whose phylogenetic relationship to Native American populations has been well worked out (*Russia_MA1_24000BP*, *Onge*, and *Mbuti*), the model is a poor fit (maximum mismatch of observed and expected *f-*statistics of |Z| = 4.8) (Figure S6B). This implies that the model of Scheib et al. (2018) does not capture some important features of the history relating these populations, and suggests that we may not be able to rely on the inferred proportions of ancestry.

If Scheib et al. (2018) were correct that there was widespread *ANC-B* ancestry in Central and South America, then *Canada_Lucier_4800BP-500BP* would not be an outgroup to *Anzick-1* and all Central and South Americans; that is, statistics of the form *f*_*4*_*(USR1, Canada_Lucier_4800BP-500BP; Anzick-1, Test Central or South America)* would often be positive. In fact, *Canada_Lucier_4800BP-500BP* is consistent with being an outgroup to all Central and South America in our analysis, as statistics of the form *f*_*4*_*(USR1, Canada_Lucier_4800BP-500BP; Anzick-1, Test Central or South America)* are all consistent with zero except for the special *Late Central Andes* individuals (as we describe elsewhere, this signal could be explained either by less than 2% *Canada_Lucier_4800BP-500BP* admixture into the *Late Central Andes* groups, or alternatively *USA_SanNicolas_1400BP*-related admixture into *Canada_Lucier_4800BP-500BP*) (modern South Americans such as *Piapoco* and *Quechua* had statistics consistent with zero as well) (Table S4). This is in line with Figure S13 of Scheib et al. (2018), where *Canada_Lucier_4800BP-500BP* is also fit as an outgroup to Central and South Americans; the fit of Figure S13 of their study is reasonable, with the maximum mismatch between observed and expected *f-*statistics being |Z| = 2.0, which is not surprising after correcting for the number of hypotheses tested.

To obtain some insight into why models such as Figure 2A of Scheib et al. (2018) could fit the data even while statistics like *f*_*4*_*(USR1, Canada_Lucier_4800BP-500BP; Anzick-1, Test Central or South America)* are for the most part consistent with being zero, we estimated the genetic drift along the edge leading to *Canada_Lucier_4800BP-500BP* that mixed into South Americans in Figure 2A. We found that it is not significantly different from zero in any of the graphs that we analyzed (Figures S6A–S6D; STAR Methods), meaning the ancestry on the *Canada_Lucier_4800BP-500BP* branch that mixes into the South American groups does not share a significant amount of genetic drift with *Canada_Lucier_4800BP-500BP* and there is no need to propose widespread mixing between *ANC-A* and *ANC-B*.

A supporting piece of evidence cited by Scheib et al. (2018) in favor of mixture between *ANC-A* and *ANC-B* lineages in Central and South Americans is that they identify present-day *Pima* and *Surui* haplotypes that match *Anzick-1* haplotypes (as a representative of *ANC-A*) more closely than *CK-13* (as a representative of *Canada_Lucier_4800BP-500BP*), and vice versa. However, Native American populations (like all human populations) have a large proportion of shared ancestral haplotypes, and incomplete lineage sorting means that even if two populations are not most closely related, in some sections of the genome they will be most closely related on a haplotypic basis. Thus, it is not clear to us that this analysis demonstrates that *Pima* and *Surui* derive from *ANC-A/ANC-B* mixtures.

In conclusion, given that *Canada_Lucier_4800BP-500BP* is consistent with being an outgroup to nearly all Central and South Americans based on *f*-statistic analysis (with the exception of the special *Late Central Andes* populations), and that there is no compelling haplotype-based evidence for *ANC-A* and *ANC-B* admixture in the history of Central and South Americans, the genetic data are in fact consistent with the scenario in which an *ANC-A* population was the sole contributor to southern branch (Central and South American populations). Thus, our results are consistent with the originally suggested null hypothesis of entirely *ANC-A* ancestry leading to Central and South Americans (Raghavan et al., 2015, Rasmussen et al., 2014, Reich et al., 2012).

To build the admixture graph shown in Figure 3, we used a skeleton graph from previous publications (Lipson and Reich, 2017, Skoglund et al., 2015). We added in groups based on previous findings (e.g., the Ancient Beringian *USR1* as an outgroup [Moreno-Mayar et al., 2018a] and the split between *ANC-A* and *ANC-B* [Reich et al., 2012, Scheib et al., 2018]). We then added additional groups new to this study using guidance from other results such as the outgroup-*f*_*3*_ matrix-based neighbor-joining tree. We stopped building the admixture graph once we had fit as many representative ancient individuals as possible that could fit without strong evidence of mixture (worst Z-score outlier f_4_ (*Han*, *USA_SanNicolas_4900BP; Argentina_ArroyoSeco2_7700BP, Canada_Lucier_4800BP-500BP)* Z = 2.9).

To build the complex admixture graphs shown in Figures 4 and 5, we used two approaches. For Figure 4, we started with the admixture graph of Figure 3, and then grafted onto it admixture events motivated by our *qpWave* results, namely mixture from an *Anzick-1*-related lineage into the earliest Chilean individual and some of the Brazil and Argentina groups, and mixture of *USA_SanNicolas_4900BP*-related ancestry into *Late Central Andes* groups. We compared models with and without admixture edges and used the model with an extra admixture edge if it decreased the maximum Z score by over 0.3.

For Figure 5 we carried out a semi-automated search in which we began with a skeleton model including all non-Native Americans and *USA_USR1_11400BP*, and then iteratively added as many other populations as we could in a greedy approach, first as simple clades in order to minimize graph complexity, and then as 2-way mixtures if the sample clade approach did not fit. Thus, for *N* populations, we first fit graphs of *m* populations and then considered all remaining *N*-*m* populations as candidates to be grafted in all fitting models with *m* populations. Each grafted population was either placed anywhere on the graph (or its two components in case of mixture were placed anywhere on the graph). This approach is described in more detail in Lazaridis et al. (2018).

The two admixture graphs shown in Figures 4 and 5 have many qualitative points of agreement including: i) *USA_USR1_11400BP* as an outgroup to all other Native Americans, ii) a split of *ANC-A* and *ANC-B* such that *ANC-B* had minimal genetic influence on all South Americans, iii) A rapid radiation of the earliest South Americans, with the earliest South Americans having very little drift on the lineages separating them, iv) distinctive shared ancestry between *Brazil_LapaDoSanto_9600BP* and *Chile_LosRieles_10900BP* on the one hand and *USA_Anzick-1_12800BP* on the other, v) distinctive shared ancestry between *USA_Anzick-1_12800BP* and *USA_SanNicolas_4900BP*, and vi) mixture of a source of ancestry with distinct relatedness to North Americans into *Late Central Andes* groups.

The primary disagreement between the admixture graphs concerns the question of whether or not *USA_Anzick_12800BP* is admixed.

In Figure 4 *USA_Anzick_12800BP* is modeled as unadmixed, and ancestry related to this group mixes into some of the Brazil, Chile, and Argentina groups as well as into *USA_SanNicolas_4900BP*. The ancestry sources can be interpreted as resulting from North to South America spreads in successive streams. There are an initial two streams from an *Anzick-1*-related group retained in *Chile_LosRieles_10900BP*, *Brazil_LapaDoSanto_9600BP*, and *Argentina_ArroyoSeco2_7700BP* and another ancestry stream that is pervasive throughout ancient South America (we cannot resolve the order of these two streams). There is a third ancestry source contributing to *Late Central Andes* groups, and a fourth ancestry source that corresponds to the *Population Y* signal in *Karitiana* and *Surui* but that we do not specifically model in the graph.

In Figure 5, most South Americans can be modeled as a mixture of a lineage that split into regional branches in Peru (*Lauricocha_8600BP* and *Cuncaicha_9000BP*), the Southern Cone (*Argentina_ArroyoSeco2_7700BP* and *Chile_LosRieles_5100BP*), and *Brazil_LapaDoSanto_9600BP*, with the lineage more closely related to *Brazil_LapaDoSanto_9600BP* then mixing into the shared ancestors of *USA_Anzick_12800BP* and *USA_SanNicolas_4900BP* (possibly reflecting a back-flow from South to North America, although, alternatively, all the splits could have occurred in North America). The model also specifies more recent admixture into *Late Central Andes* population of a lineage with a distinctive relatedness to North Americans (this model also included West Eurasian related admixture in *Canada_Lucier_4800BP-500BP* that likely reflects a low level of contamination in these samples).

Both models shown in Figures 4 and 5 are reasonable statistical fits (maximum Z-scores of 3.4 and 2.9 with only transitions in CpG sites removed, and 3.0 and 2.9 when all transitions are removed), and we were unable to resolve which was better. Additional sampling of early North and South Americans could help to resolve the true model.

In Figure S5, we present various modifications of these models, including some that add *Surui* which has evidence of a fourth source of “*Population Y*” ancestry that bears a different relationship to Asians.

#### Analyses of phenotypically relevant SNPs

We analyzed sequences at SNPs previously known to be relevant to interesting phenotypic traits. We used *samtools* version 1.3.1 (Li, 2011, Li et al., 2009) *mpileup* with the settings -d 8000 -B -q30 -Q30 to obtain information about each read from the bam files of our samples. We used the fasta file from human genome GRCh37 (hg19) for the pileup. We counted the number of derived and ancestral variants at each analyzed position using a custom Python script.

Besides *EDAR* we analyzed several other phenotypically relevant variants including one variant *TBX15* which affects body fat distribution (Racimo et al., 2017), a variant in *SLC16A11*, which predisposes individuals to diabetes (Williams et al., 2014), 2 variants in *NOS3* and *EGLN1* believed to facilitate life at high altitudes (Fehren-Schmitz and Georges, 2016), the top 10 variants from a recent study on natural selection in Andeans (Crawford et al., 2017), and a variant at the fatty acid desaturase gene *FADS2* with evidence of natural selection (Amorim et al., 2017).

We observed that the *SLC16A11* variant rs13342232 is homozygous ancestral (A) in *USR1* and *Anzick-1*, but the derived allele is present in 17/32 of the individuals that had coverage at the variant, which is approximately the frequency observed in present day Native Americans (we observe no significant correlation with time or location).

The *TBX15* allele is heterozygous in *USR1* but homozygous derived (A) in *Anzick-1* and all other individuals with at least one sequence covering the SNP (39) except 2 *Belize_SakiTzul_7400BP* and a present-day *Mayan* individual that were heterozygous, and 1 *Argentina-Chile_FuegoPatagonian_100BP* that had a single sequence supporting the ancestral position. This reflects the approximate allele frequency present in present day Native Americans (Racimo et al., 2017), meaning that selection, if it occurred, likely did so prior to the diversification of Native Americans.

The *NOS3* and *EGLN1* derived and ancestral alleles were present in appreciable frequencies in all time periods and locations, and we lacked enough samples to assess whether a prior report of selection on these variants (Fehren-Schmitz and Georges, 2016) was consistent with our data.

As expected, all individuals were homozygous for the ancestral allele at *SLC45A2* (C) and *LCT* (G), and *SLC24A5* (G), indicating darker skin color and lack of lactase persistence.

In the Greenland Inuit *FADS2* has been shown to have experienced selection related to cold adaptation and to a diet rich of proteins (Fumagalli et al., 2015). Selection scans in non-Arctic Native American groups who share a substantial proportion of their ancestry with the Inuit also identified the *FADS2* locus as being under positive selection, and it has been proposed that the adaptation took place in a common ancestral group before their entrance in the Americas (Amorim et al., 2017). All our ancient individuals harbor the derived variant of a *FADS2* SNP (chr11:pos61597212, rs174570) supporting the view that the selection could have taken place in the ancestral population of Native Americans (Table S6).

#### Insights into more recent history of Brazil based on Jabuticabeira 2 individuals

Maritime societies are documented on the coast of southern and southeastern Brazil since about 8000 BP. Even without agriculture and pottery, these groups achieved impressive demographic densities. Their most outstanding cultural practice was the building of hundreds of shell-mounds, some of monumental magnitude, that were used as a funerary ground (up to one thousand skeletons are estimated to be included in mounds that could be 50 m high and 200 m in diameter). Analysis of sex-biased morphological variation suggest these groups were matrilocal (Hubbe et al., 2009). It is still not clear whether shell-mound builders constituted a pan-regional society with a single origin sharing ancestry and language, or if they were groups of independent origins who adopted a similar subsistence strategy focused on a maritime economy and the construction of shell mounds. Nevertheless, around 2000-1000 BP there is a clear decline and eventually cessation of shell-mound construction. A highly debated question in South American pre-colonial history concerns the nature of the disappearance of the Sambaqui archaeological tradition and the role in this process of two main population movements in the region – arrival of Ge speakers and arrival of Tupi speakers, hypothesized to have occurred at ∼2000 BP and ∼1000 BP, respectively.

While it has been proposed that Sambaqui shell mound builders and proto-Ge speakers probably interacted, the arrival of Tupi-Guarani speakers (such as present-day *Parakana* and *Guarani*) is hypothesized to have involved a rather abrupt population replacement leading to the complete disappearance of the Sambaqui culture – represented in the current study by Jabuticabeira 2 (Hubbe et al., 2009, Iriarte et al., 2017).

Our analyses of the genetic affinities of *the Brazil_Jabuticabeira2_2000BP* individuals to modern groups provides the first genetic evidence to test this model. In Figure S3 it appears that the *Kaingang* (a Ge speaking group from southern Brazil) and the *Arara* (a Carib speaking group part of the Ge-Pano-Carib family from northern Brazil) have a greater genetic affinity with *Brazil_Jabuticabeira2_2000BP* than do groups that speak Tupi-Guarani languages. This pattern is confirmed with statistics of the form *f*_*4*_*(Mbuti, Brazil_Jabuticabeira2_2000BP; Guarani, Kaingang)* and *f*_*4*_*(Mbuti, Brazil_Jabuticabeira2_2000BP; Parakana, Arara)* that are significantly positive (Z scores of 2.7 and 3.2, respectively, despite fewer than 50,000 SNPs being available for analysis) (Table S1), suggesting that present-day Ge speakers across a wide geographic region harbor specific affinities to Sambaqui shell mound builders, consistent with some elements of shared ancestry in Ge speakers. This pattern holds even when we remove the most recent Jabuticabeira 2 individual dated to ∼1200BP (Table S1).

Within Jabuticabeira 2, there are two distinct periods of occupation. The earliest is dated to 2500-2200 BP and can be considered a late expression of the classic Sambaqui phenomenon that first appears in the region around 8000 BP. The later occupation event is dated to around 1500 BP and archaeologically is expressed by the formation of layers of dark earth on top of the shell-mounds. This transformation is not restricted to Jabuticabeira 2 as it has been documented at several other locations along the Atlantic coast. Possible factors to explain this transformation range from environmental changes (e.g., total depletion of mollusk banks) to the arrival of new people or simply the implementation of new practices. In the present study the only individual of Jabuticabeira 2 coming from the second event of occupation is Burial 102 - all others are from the classic Sambaqui phase of occupation. Our genome-wide data are thin for this individual and lack the resolution to test for differences in ancestry between the groups. However, mtDNA sequences of all seven individuals from the classic Sambaqui horizon share the same haplogroup C1c indicating low mtDNA diversity, while Burial 102 carries mtDNA haplogroup B2. More individuals from the later period of occupation of the site should be able to reveal if Burial 102 is representative of a population shift.

### Data and Software Availability

Raw sequences (bam files) from the 49 newly reported ancient individual with genome-wide data and 48 newly reported individuals with mtDNA data are available from the European Nucleotide Archive. The accession number for the sequence data reported in this paper is ENA: PRJEB28961.

## Supplemental Information

Table S1. Relatedness of Ancient to Present-Day People, Related to Figures 2, S1, and S2 *f*_*4*_-statistics on the Illumina dataset of the form *f*_*4*_*(Mbuti, Test; South American 1, South American 2)* where “Test” is a newly reported ancient group, “South American 1” is the population with highest affinity to *Test* in outgroup *f*_*3*_*-*statistics (excluding *Chorotega* due to limited data), and “South American 2” is the population from the Central Andes (*Quechua*), Southern Cone (*Chilote/Huilliche*) or Amazonia (*Karitiana*) that produces the most negative Z score. Because both “Highest outgroup *f*_*3*_” and “South America” are selected to maximize Z-scores, there is a multiple hypothesis issue that we do not account for here, and the true significance is less than would be expected from the absolute Z-score. Specifically, we are examining 50 comparison populations for each *Test* population to identify the one that maximizes the outgroup-*f*_*3*_ statistic, and 3 representative South American populations, for a total of 150 tests (Related to Figure S1). We also report *f*_*4*_-statistics on the Illumina dataset of the form *f*_*4*_*(Mbuti, Brazil_Jabuticabeira2_2000BP; Modern Brazil 1, Modern Brazil 2)*, and on the *Brazil_Jabuticabeira2_subset* data, which are the same as *Brazil_Jabuticabeira2_2000BP* data except with the 1200BP individual removed (I9056_d).

Table S2. Affinity of Early South Americans to North Americans, Related to Table S4 Representative f_4_-statistics on 1240K dataset of the form f_4_(Mbuti, Anzick-1; Early South American, Brazil_LapaDoSanto_9600BP or Late Central Andes) or f_4_(Mbuti, Canada_Lucier_4800BP-500BP; Brazil_LapaDoSanto_9600BP or Brazil_Laranjal_6700BP, Late or Modern Peruvian or Chilean).

Table S3. Data Description, Ancient DNA Workflow, mtDNA, Radiocarbon Dates, Related to Figure 1

Table S4. *f*_*4*_*-* and *f*_*3*_*-*Statistics, Related to Figures 1, 4, 5, S5D–S5F, and S6A–S6D and Table S2

Table S5. *qpWave* Analyses, Related to Figures 4 and S4

Table S6. *Population Y* Signal Analyses, Related to Figures S5G–S5I

Table S7. Phenotypic Allele Analyses, Related to STAR Methods
