# Supplementary material for: Reconstructing the Deep Population History of Central and South America
Source: Cell. 2018 Nov 15;175(5):1185–1197.e22. doi: 10.1016/j.cell.2018.10.027 (PMC6327247; doi:10.1016/j.cell.2018.10.027)
Supplement: Table S1. Relatedness of Ancient to Present-Day People, Related to Figures 2, S1, and S2 — f4-statistics on the Illumina dataset of the form f4(Mbuti, Test; South American 1, South American 2) where “Test” is a newly reported ancient group, “South American 1” is the population with highest affinity to Test in outgroup f3-statistics (excluding Chorotega due to limited data), and “South American 2” is the population from the Central Andes (Quechua), Southern Cone (Chilote/Huilliche) or Amazonia (Karitiana) that produces the most negative Z score. Because both “Highest outgroup f3” and “South America” are selected to maximize Z-scores, there is a multiple hypothesis issue that we do not account for here, and the true significance is less than would be expected from the absolute Z-score. Specifically, we are examining 50 comparison populations for each Test population to identify the one that maximizes the outgroup-f3 statistic, and 3 representative South American populations, for a total of 150 tests (Related to Figure S1). We also report f4-statistics on the Illumina dataset of the form f4(Mbuti, Brazil_Jabuticabeira2_2000BP; Modern Brazil 1, Modern Brazil 2), and on the Brazil_Jabuticabeira2_subset data, which are the same as Brazil_Jabuticabeira2_2000BP data except with the 1200BP individual removed (I9056_d). [file mmc1.pdf]

Cell, Volume *175*

## Supplemental Information

# Reconstructing the Deep Population History of Central and South America

**Cosimo Posth, Nathan Nakatsuka, Iosif Lazaridis, Pontus Skoglund, Swapan Mallick, Thiseas C. Lamnidis, Nadin Rohland, Kathrin Nägele, Nicole Adamski, Emilie Bertolini, Nasreen Broomandkhoshbacht, Alan Cooper, Brendan J. Culleton, Tiago Ferraz, Matthew Ferry, Anja Furtwängler, Wolfgang Haak, Kelly Harkins, Thomas K. Harper, Tábita Hünemeier, Ann Marie Lawson, Bastien Llamas, Megan Michel, Elizabeth Nelson, Jonas Oppenheimer, Nick Patterson, Stephan Schiffels, Jakob Sedig, Kristin Stewardson, Sahra Talamo, Chuan-Chao Wang, Jean-Jacques Hublin, Mark Hubbe, Katerina Harvati, Amalia Nuevo Delaunay, Judith Beier, Michael Francken, Peter Kaulicke, Hugo Reyes-Centeno, Kurt Rademaker, Willa R. Trask, Mark Robinson, Said M. Gutierrez, Keith M. Prufer, Domingo C. Salazar-García, Eliane N. Chim, Lisiane Müller Plumm Gomes, Marcony L. Alves, Andersen Liryo, Mariana Inglez, Rodrigo E. Oliveira, Danilo V. Bernardo, Alberto Barioni, Veronica Wesolowski, Nahuel A. Scheifler, Mario A. Rivera, Claudia R. Plens, Pablo G. Messineo, Levy Figuti, Daniel Corach, Clara Scabuzzo, Sabine Eggers, Paulo DeBlasis, Markus Reindel, César Méndez, Gustavo Politis, Elsa Tomasto-Cagigao, Douglas J. Kennett, André Strauss, Lars Fehren-Schmitz, Johannes Krause, and David Reich**

**Table S1. Relatedness of ancient to present-day people.** $f_4$-statistics on the Illumina dataset of the form $f_4$*(Mbuti, Test; South American 1, South American 2)* where "Test" is a newly reported ancient group, "South American 1" is the population with highest affinity to *Test* in outgroup $f_3$-statistics (excluding *Chorotega* due to limited data), and "South American 2" is the population from the Central Andes (*Quechua*), Southern Cone (*Chilote/Huilliche*) or Amazonia (*Karitiana*) that produces the most negative Z score. Because both "Highest outgroup $f_3$" and "South America" are selected to maximize Z-scores, there is a multiple hypothesis issue that we do not account for here, and the true significance is less than would be expected from the absolute Z-score. Specifically, we are examining 50 comparison populations for each *Test* population to identify the one that maximizes the outgroup-$f_3$ statistic, and 3 representative South American populations, for a total of 150 tests (Related to Figure S1). We also report $f_4$-statistics on the Illumina dataset of the form $f_4$*(Mbuti, Brazil_Jabuticabeira2_2000BP; Modern Brazil 1, Modern Brazil 2)*, and on the *Brazil_Jabuticabeira2_subset* data, which are the same as *Brazil_Jabuticabeira2_2000BP* data except with the 1200BP individual removed (I9056_d) (Related to Figure 2 and Figures S1 and S2).

| Outgroup | Test | South American 1 (Region or Language Family) | South American 2 (Region or Language Family) | $f_4$-statistic | Z-score | Number of SNPs |
|---|---|---|---|---|---|---|
| *Mbuti* | *Chile_Conchali_700BP* | *Huilliche (Southern Cone)* | *Quechua (Central Andes)* | -0.005311 | -15.441 | 280709 |
| *Mbuti* | *Peru_Laramate_900BP* | *Quechua (Central Andes)* | *Karitiana (Amazonia)* | -0.004056 | -14.577 | 307061 |
| *Mbuti* | *Chile_PicaOcho_700BP* | *Aymara (Central Andes)* | *Karitiana (Amazonia)* | -0.003705 | -9.321 | 157160 |
| *Mbuti* | *Peru_Cuncaicha_4200BP* | *Aymara (Central Andes)* | *Karitiana (Amazonia)* | -0.003619 | -9.128 | 272057 |
| *Mbuti* | *Chile_LosRieles_5100BP* | *Huilliche (Southern Cone)* | *Quechua (Central Andes)* | -0.00364 | -8.672 | 241676 |
| *Mbuti* | *Peru_Lauricocha_8600BP* | *Quechua (Central Andes)* | *Karitiana (Amazonia)* | -0.002728 | -8.24 | 305918 |
| *Mbuti* | *Peru_Cuncaicha_3300BP* | *Aymara (Central Andes)* | *Karitiana (Amazonia)* | -0.003143 | -7.907 | 236585 |
| *Mbuti* | *Peru_LaGalgada_4100BP* | *Quechua (Central Andes)* | *Karitiana (Amazonia)* | -0.002808 | -7.651 | 285857 |
| *Mbuti* | *Peru_Lauricocha_3500BP* | *Quechua (Central Andes)* | *Karitiana (Amazonia)* | -0.002953 | -7.471 | 189510 |
| *Mbuti* | *Argentina_ArroyoSeco2_7700BP* | *Chono (Southern Cone)* | *Quechua (Central Andes)* | -0.002334 | -5.767 | 278258 |
| *Mbuti* | *Peru_Cuncaicha_9000BP* | *Aymara (Central Andes)* | *Karitiana (Amazonia)* | -0.002357 | -5.575 | 276660 |
| *Mbuti* | *Peru_Lauricocha_5800BP* | *Quechua (Central Andes)* | *Chilote (Southern Cone)* | -0.00254 | -5.5 | 192658 |
| *Mbuti* | *Argentina_LagunaChica_6800BP* | *Chono (Southern Cone)* | *Quechua (Central Andes)* | -0.001851 | -3.663 | 130251 |
| *Mbuti* | *Chile_LosRieles_10900BP* | *Chono (Southern Cone)* | *Huilliche (Southern Cone)* | -0.001817 | -3.128 | 228506 |
| *Mbuti* | *Brazil_Moraes_5800BP* | *Arara (Amazonia)* | *Huilliche (Southern Cone)* | -0.002545 | -2.977 | 82759 |
| *Mbuti* | *Brazil_Laranjal_6700BP* | *Arara (Amazonia)* | *Chilote (Southern Cone)* | -0.001435 | -2.171 | 159697 |
| *Mbuti* | *Belize_MayahakCabPek_9300BP* | *Jamamadi (Amazonia)* | *Karitiana (Amazonia)* | -0.001341 | -1.787 | 137431 |
| *Mbuti* | *Brazil_LapaDoSanto_9600BP* | *Arara (Amazonia)* | *Karitiana (Amazonia)* | -0.00086 | -1.919 | 298748 |
| *Mbuti* | *Belize_SakiTzul_7400BP* | *Arara (Amazonia)* | *Huilliche (Southern Cone)* | -0.001036 | -1.543 | 164954 |

| | | | | | | |
|---|---|---|---|---|---|---|
| *Mbuti* | *Brazil_Jabuticabeira2_2000BP* | *Guarani (Tupi-Guarani)* | *Kaingang (Ge)* | 0.002102 | 2.687 | 45181 |
| *Mbuti* | *Brazil_Jabuticabeira2_2000BP* | *Parakana (Tupi-Guarani)* | *Kaingang (Ge)* | 0.003853 | 3.842 | 44322 |
| *Mbuti* | *Brazil_Jabuticabeira2_2000BP* | *Arara (Carib)* | *Kaingang (Ge)* | 0.000202 | 0.185 | 42046 |
| *Mbuti* | *Brazil_Jabuticabeira2_2000BP* | *Guarani (Tupi-Guarani)* | *Arara (Carib)* | 0.001536 | 1.815 | 49171 |
| *Mbuti* | *Brazil_Jabuticabeira2_2000BP* | *Parakana (Tupi-Guarani)* | *Arara (Carib)* | 0.00342 | 3.155 | 48341 |
| *Mbuti* | *Brazil_Jabuticabeira2_subset* | *Guarani (Tupi-Guarani)* | *Kaingang (Ge)* | 0.001771 | 2.155 | 40713 |
| *Mbuti* | *Brazil_Jabuticabeira2_subset* | *Parakana (Tupi-Guarani)* | *Kaingang (Ge)* | 0.003323 | 3.124 | 39931 |
| *Mbuti* | *Brazil_Jabuticabeira2_subset* | *Arara (Carib)* | *Kaingang (Ge)* | -0.000132 | -0.114 | 37881 |
| *Mbuti* | *Brazil_Jabuticabeira2_subset* | *Guarani (Tupi-Guarani)* | *Arara (Carib)* | 0.001523 | 1.718 | 44249 |
| *Mbuti* | *Brazil_Jabuticabeira2_subset* | *Parakana (Tupi-Guarani)* | *Arara (Carib)* | 0.003194 | 2.759 | 43489 |
