# Supplementary material for: Reconstructing the Deep Population History of Central and South America
Source: Cell. 2018 Nov 15;175(5):1185–1197.e22. doi: 10.1016/j.cell.2018.10.027 (PMC6327247; doi:10.1016/j.cell.2018.10.027)
Supplement: Table S2. Affinity of Early South Americans to North Americans, Related to Table S4 — Representative f4-statistics on 1240K dataset of the form f4(Mbuti, Anzick-1; Early South American, Brazil_LapaDoSanto_9600BP or Late Central Andes) or f4(Mbuti, Canada_Lucier_4800BP-500BP; Brazil_LapaDoSanto_9600BP or Brazil_Laranjal_6700BP, Late or Modern Peruvian or Chilean). [file mmc2.pdf]

**Table S2. Affinity of Early South Americans to North Americans.** Representative $f_4$-statistics on 1240K dataset of the form *$f_4$(Mbuti, Anzick-1; Early South American, Brazil_LapaDoSanto_9600BP or Late Central Andes)* or *$f_4$(Mbuti, Canada_Lucier_4800BP-500BP; Brazil_LapaDoSanto_9600BP or Brazil_Laranjal_6700BP, Late or Modern Peruvian or Chilean).* Related to Table S4.

| Outgroup | North America | Early South American | Other | $f_4$-statistic | Z-score | Number of SNPs |
|---|---|---|---|---|---|---|
| *Mbuti* | *USA_Anzick-1_12800BP* | *Peru_Lauricocha_8600BP* | *Chile_ LosRieles_10900BP* | 0.002834 | 3.425 | 762,120 |
| *Mbuti* | *USA_Anzick-1_12800BP* | *Peru_Cuncaicha_9000BP* | *Chile_LosRieles_10900BP* | 0.002241 | 2.477 | 726,818 |
| *Mbuti* | *USA_Anzick-1_12800BP* | *Argentina_ArroyoSeco2_7700BP* | *Chile_ LosRieles_10900BP* | 0.001552 | 2.020 | 754,332 |
| *Mbuti* | *USA_Anzick-1_12800BP* | *Peru_Lauricocha_8600BP* | *Brazil_LapaDoSanto_9600BP* | 0.001633 | 3.109 | 955,886 |
| *Mbuti* | *USA_Anzick-1_12800BP* | *Peru_Cuncaicha_9000BP* | *Brazil_LapaDoSanto_9600BP* | 0.001005 | 1.542 | 875,139 |
| *Mbuti* | *USA_Anzick-1_12800BP* | *Argentina_ArroyoSeco2_7700BP* | *Brazil_LapaDoSanto_9600BP* | 0.000384 | 0.803 | 932,942 |
| *Mbuti* | *USA_Anzick-1_12800BP* | *Peru_Lauricocha_8600BP* | *Peru_Cuncaicha_4200BP* | 0.001944 | 3.007 | 834,310 |
| *Mbuti* | *USA_Anzick-1_12800BP* | *Peru_Cuncaicha_9000BP* | *Peru_Cuncaicha_4200BP* | 0.001489 | 1.989 | 794,865 |
| *Mbuti* | *USA_Anzick-1_12800BP* | *Argentina_ArroyoSeco2_7700BP* | *Peru_Cuncaicha_4200BP* | 0.000749 | 1.197 | 823,581 |
| *Mbuti* | *Canada_Lucier_4800BP-500BP* | *Brazil_LapaDoSanto_9600BP* | *Peru_Quechua_Modern* | 0.001442 | 3.290 | 204,960 |
| *Mbuti* | *Canada_Lucier_4800BP-500BP* | *Brazil_Laranjal_6700BP* | *Peru_Quechua_Modern* | 0.002161 | 3.429 | 116,092 |
| *Mbuti* | *Canada_Lucier_4800BP-500BP* | *Brazil_LapaDoSanto_9600BP* | *Peru_Aymara_Modern* | 0.00149 | 2.445 | 208,965 |
| *Mbuti* | *Canada_Lucier_4800BP-500BP* | *Brazil_Laranjal_6700BP* | *Peru_Aymara_Modern* | 0.001889 | 2.336 | 118,118 |
| *Mbuti* | *Canada_Lucier_4800BP-500BP* | *Brazil_LapaDoSanto_9600BP* | *Peru_LaGalgada_4100BP* | 0.002109 | 3.567 | 178,534 |
| *Mbuti* | *Canada_Lucier_4800BP-500BP* | *Brazil_Laranjal_6700BP* | *Peru_LaGalgada_4100BP* | 0.002678 | 3.262 | 108,672 |
| *Mbuti* | *Canada_Lucier_4800BP-500BP* | *Brazil_LapaDoSanto_9600BP* | *Peru_Laramate_900BP* | 0.001158 | 2.606 | 192,122 |
| *Mbuti* | *Canada_Lucier_4800BP-500BP* | *Brazil_Laranjal_6700BP* | *Peru_Laramate_900BP* | 0.002455 | 3.917 | 113,762 |
| *Mbuti* | *Canada_Lucier_4800BP-500BP* | *Brazil_LapaDoSanto_9600BP* | *Chile_PicaOcho_700BP* | 0.000528 | 0.722 | 101,447 |
| *Mbuti* | *Canada_Lucier_4800BP-500BP* | *Brazil_Laranjal_6700BP* | *Chile_PicaOcho_700BP* | 0.001542 | 1.617 | 69,706 |
| *Mbuti* | *Canada_Lucier_4800BP-500BP* | *Brazil_LapaDoSanto_9600BP* | *Chile_Conchali_700BP* | 0.000924 | 1.796 | 179,370 |
| *Mbuti* | *Canada_Lucier_4800BP-500BP* | *Brazil_Laranjal_6700BP* | *Chile_Conchali_700BP* | 0.00201 | 2.739 | 109,689 |
| *Mbuti* | *Canada_Lucier_4800BP-500BP* | *Brazil_LapaDoSanto_9600BP* | *Peru_Cuncaicha_4200BP* | 0.001019 | 1.594 | 173,982 |
| *Mbuti* | *Canada_Lucier_4800BP-500BP* | *Brazil_Laranjal_6700BP* | *Peru_Cuncaicha_4200BP* | 0.002244 | 2.617 | 107,094 |
| *Mbuti* | *Canada_Lucier_4800BP-500BP* | *Brazil_LapaDoSanto_9600BP* | *Peru_Cuncaicha_3300BP* | 0.000325 | 0.495 | 155,599 |
| *Mbuti* | *Canada_Lucier_4800BP-500BP* | *Brazil_Laranjal_6700BP* | *Peru_Cuncaicha_3300BP* | 0.00074 | 0.853 | 98,694 |
